# SARS-CoV-2 Evolution: Implications for Diagnosis, Treatment, Vaccine Effectiveness and Development

**DOI:** 10.3390/vaccines13010017

**Published:** 2024-12-28

**Authors:** Fabrizio Angius, Silvia Puxeddu, Silvio Zaimi, Serena Canton, Sepehr Nematollahzadeh, Andrea Pibiri, Ilenia Delogu, Gualtiero Alvisi, Meng Ling Moi, Aldo Manzin

**Affiliations:** 1 Microbiology and Virology Unit, Department of Biomedical Sciences, University of Cagliari, University Campus, 09042 Monserrato, Italyandrea.pibiri95@gmail.com (A.P.); ilenia.delogu@unica.it (I.D.); aldo.manzin@unica.it (A.M.); 2 Department of Molecular Medicine, University of Padova, 35121 Padova, Italy; sepehr.nematollahzadeh@studenti.unipd.it (S.N.); gualtiero.alvisi@unipd.it (G.A.); 3 School of International Health, Graduate School of Medicine, The University of Tokyo, Tokyo 113-0033, Japan

**Keywords:** SARS-CoV-2, variants, vaccines, COVID-19, diagnosis, treatments

## Abstract

The COVID-19 pandemic, driven by the rapid evolution of the SARS-CoV-2 virus, presents ongoing challenges to global public health. SARS-CoV-2 is characterized by rapidly evolving mutations, especially in (but not limited to) the spike protein, complicating predictions about its evolutionary trajectory. These mutations have significantly affected transmissibility, immune evasion, and vaccine efficacy, leading to multiple pandemic waves with over half a billion cases and seven million deaths globally. Despite several strategies, from rapid vaccine development and administration to the design and availability of antivirals, including monoclonal antibodies, already having been employed, the persistent circulation of the virus and the emergence of new variants continue to result in high case numbers and fatalities. In the past four years, immense research efforts have contributed much to our understanding of the viral pathogenesis mechanism, the COVID-19 syndrome, and the host–microbe interactions, leading to the development of effective vaccines, diagnostic tools, and treatments. The focus of this review is to provide a comprehensive analysis of the functional impact of mutations on diagnosis, treatments, and vaccine effectiveness. We further discuss vaccine safety in pregnancy and the implications of hybrid immunity on long-term protection against infection, as well as the latest developments on a pan-coronavirus vaccine and nasal formulations, emphasizing the need for continued surveillance, research, and adaptive public health strategies in response to the ongoing SARS-CoV-2 evolution race.

## 1. Introduction

**Figure 1:**
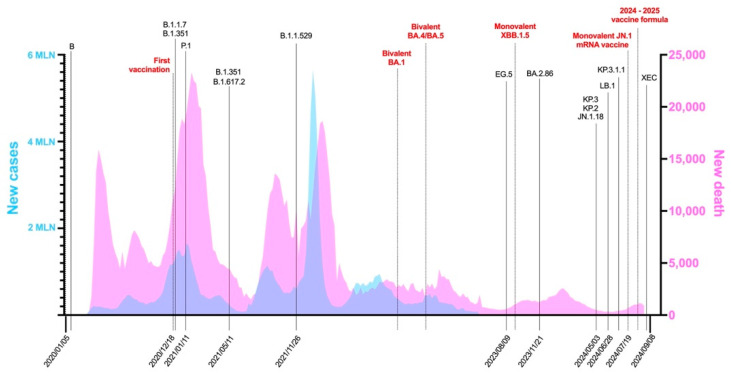
Diagram of new COVID-19 cases and deaths. The image shows new cases (blue) and deaths (pink) in the United States along a timeline. The vertical lines represent the emergence of the variants of most concern (black font) and vaccine updates available to the population (red font). Data were obtained and modified from the WHO COVID-19 Situation Reports [7].

On 31 December 2019, the WHO’s China Country Office was informed of cases of pneumonia of an unknown etiology detected in Wuhan City of Hubei Province in China [1]. The Chinese authorities identified a novel coronavirus first named nCoV-2019, which was isolated on 7 January 2020, and its genome sequence was shared globally on 12 January 2020 in the effort to develop specific diagnostics [1]. Since then, the renamed severe acute respiratory syndrome coronavirus 2 (SARS-CoV-2) has spread around the world to the point that in December 2020, cumulative cases amounted to 75 million, with 1.6 million deaths globally since the start of the pandemic (11 March 2020) [2,3]. The increase in related coronavirus disease (COVID-19) cases in the US (Figure 1) recorded in 2021 contrasts with implementing policies such as lockdowns, social distancing, mask mandates, and the rollout of vaccines. Although in the initial phase of the pandemic, restrictive measures and vaccines were demonstrated to be effective in reducing virus transmission, these policies partially failed to limit the spread of the virus, though they proved essential in containing its pathogenicity (Figure 1). The failure to contain the epidemic can be attributed to three major issues: public hostility toward vaccination campaigns, the necessity of resuming work and social activities, and the rapid evolutionary mechanisms of the virus, which enhance its fitness in a rather short period of time [4]. In fact, the genome of SARS-CoV-2 is subjected to random mutations which influence both its structural and non-structural genes [5]. As a result of this genetic variability, SARS-CoV-2 variants have emerged, causing a possible threat to public health. The genetic alterations change the viral phenotype and affect its transmissibility, virulence, and severity of clinical manifestations [6].

**Figure 2:**
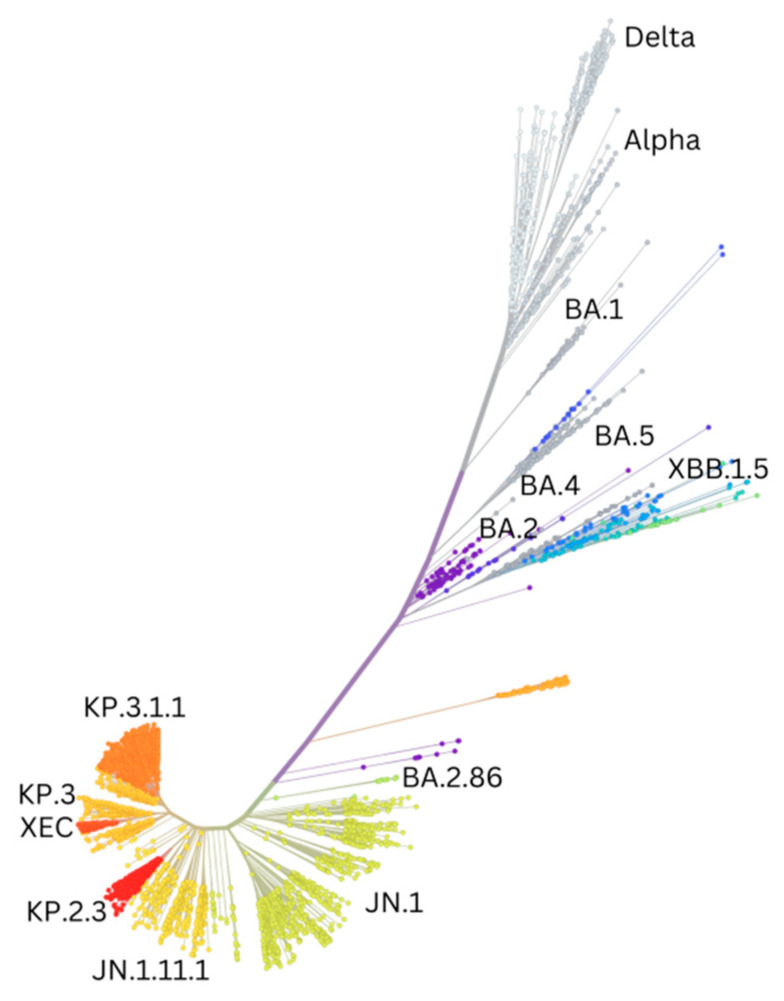
Genomic phylogeny of SARS-CoV-2 with global subsampling over the past 6 months. This figure presents an unrooted phylogenetic tree depicting the evolutionary relationships among globally circulating SARS-CoV-2 variants. The analysis incorporated viral genomic sequences sampled between December 2019 and September 2024. The phylogeny was built with the online tool nextstrain/ncov [21] and data from the GISAID database [22], updated to 15 October 2024. Variants are indicated with circles and color codes representing the different clades. Only the emerging variant names are indicated. The length of each branch represents the divergence.

**Figure 3:**
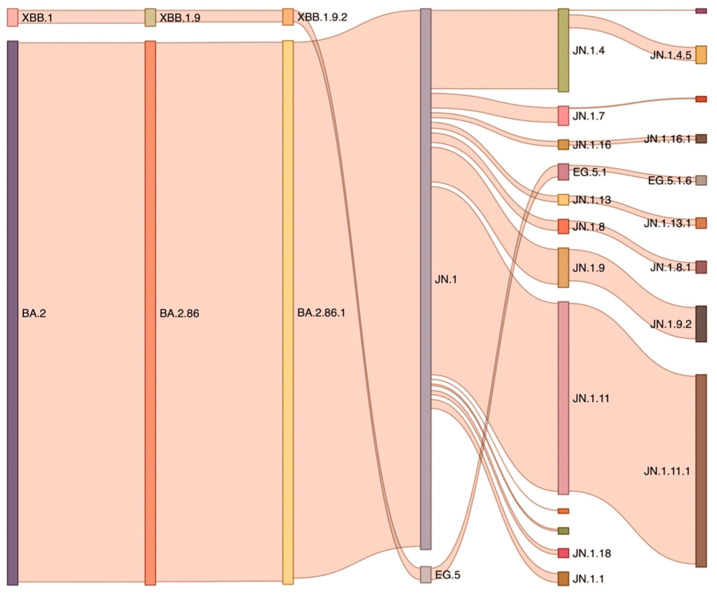
COVID-19 genomic sequencing Sankey diagram for the United States. Sankey diagram depicts the relationship between nextclade lineages observed in genomes sequenced from 1 January up to 25 September 2024 in the US (as a reference). The height of each bar represents the relative difference in the sequences for each variant. Data were obtained from gisaid.org [22] and modified by using an online tool [23].

The increase in reported cases in October and November 2021 notwithstanding, the global number of deaths remained similar to that reported in the previous period (Figure 1). This was attributed to the emergence of the Omicron variant (Table 1 and Figure 2) which due to its significant differences (Figure 3) required additional efforts in the development of new diagnostic tests, updates to the vaccination campaign, and therapeutic strategies [3]. This trend continued through 2022 [8]. During this period, Omicron remained dominant, with several subvariants emerging and circulating. Severe cases and deaths remained lower than during previous waves (Figure 1), largely due to widespread population immunity from vaccinations and prior cases [9,10] and the lower pathogenicity of Omicron. The year 2022 also saw advances in containment strategies, with the development of updated vaccines, new diagnostic tools such as at-home testing [11,12], and the introduction of antiviral treatments like Paxlovid [13,14,15]. By 5 May 2023, the WHO declared the end of the pandemic, but the year saw a further increase in new cases, accompanied by an important decrease in fatalities (Figure 1) due to the immunogenic status of the world’s population [16]. However, incidence was underestimated due to increased self-diagnosis with rapid home tests [17,18,19]. At the time of writing (October 2024), the circulation of the virus is still ongoing and remains significant, with a continuous emergence of mutations leading to new variants (Figure 3). This is evidenced by the appearance in June 2024 of the MV.1 variant (Table 1 and Figure 3), which was first detected in the Indian state of Maharashtra. This variant is drawing attention due to its rapid spread, having already reached nine countries across four continents, including the United States, Canada, and some European countries like Portugal, Ireland, and Norway [20]. This rapid spread is raising concerns, especially because MV.1 might challenge currently dominant variants such as KP.3.1.1 and XEC, potentially causing new waves of cases. Globally, MV.1 is growing 4.4% faster per day (31% per week) than the JN.1 and DeFLuQE variants while slightly trailing behind XEC’s current growth rate.

This review focuses on the genetic mutations and epidemiological implications of emerging SARS-CoV-2 variants and their significant effects on viral transmissibility and immune escape. Recent surges are linked to waning immunity and relaxed mitigation measures, although severe outcomes have decreased. However, long COVID remains a concern even in mild cases, underscoring the potential need for variant-specific vaccines and the potential of nasal vaccines to enhance mucosal immunity and reduce transmission. The impact of various variants on pregnancy outcomes and the efficacy of vaccination in minimizing adverse events are also explored. Hybrid immunity, along with its mechanisms, implications for future vaccine development, diagnostic challenges posed by viral evolution, and current treatment strategies, is examined as well.

## 2. The Close Relationship Between Mutations and Vaccines

The rise of COVID-19 marked a pivotal moment in recent history, as it caused a wave of challenges for human health and society which ended up reshaping several aspects of our world, including healthcare landscapes, societal structures, and economies. In this respect, the development of vaccines has been crucial in the global response to this threat [24,25,26]. The importance of vaccines lies in their ability to substantially reduce the risk of transmitting the disease, contracting a severe form of the illness, and death. This aspect is crucial for several reasons, foremost among which is alleviation of the burden on healthcare systems, which risked being overwhelmed during the pre-vaccine pandemic era. The reduction in transmissibility had a less evident but quite important positive effect, that being the fact that this minimized the chances of mutations which could potentially lead to more virulent variants. The development of vaccines foresees the selection of either whole or specific parts of the virus to be used as antigens for stimulating antibody production, targeting and neutralizing the virus once an infection occurs [27].

**Figure 4:**
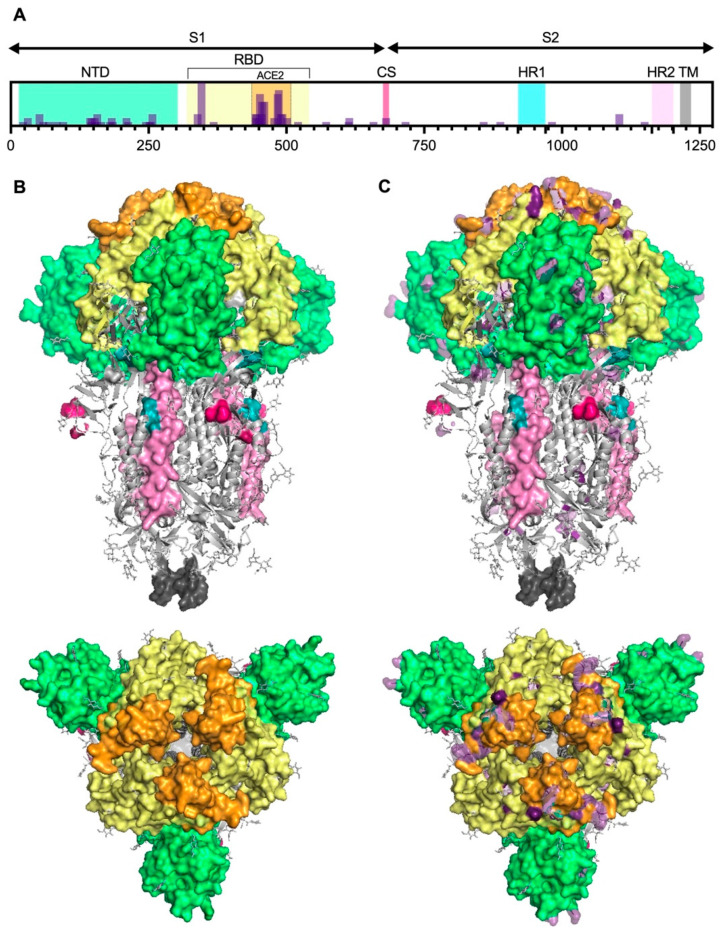
Representation of the SARS-CoV-2 S protein. (**A**) Schematic primary protein structure and (**B**) ribbon surface diagram by PyMOL (Schrödinger) using the crystal structure of the S glycoprotein in its closed state (PDB: 6VXX). The antibodies targeted the NTD and RBD with the ACE2 binding sequence, and the main protein domains are consistently indicated in different colors. Single amino acid positions involved in mutations are indicated by purple bars inside the primary structure, with the relative occurrence frequency proportionally represented by the bar height or (**C**) by different color intensities in the ribbon surface diagram. NTD = N-terminal domain; RBD = receptor-binding domain; CS = protease cleavage site; HR1 = heptad repeat 1; HR2 = heptad repeat 2; TM = transmembrane domain.

In the case of SARS-CoV-2, most approved vaccines target the S protein, which is critical for the virus’s entry into host cells. Similar to other enveloped animal riboviruses, it initiates infection by introducing its capsid and genome into the cell cytoplasm through fusion with cellular membranes. This critical step is facilitated by activation of the S protein, which is solely responsible for mediating membrane fusion. S is a trimeric class I fusion glycoprotein protruding from the viral surface and consisting of two functional subunits: S1 and S2 (Figure 4). S1 contains two domains, namely the receptor binding domain (RBD) and the N-terminal domain (NTD). The RBD directly interacts with its specific angiotensin-converting enzyme 2 (ACE2) receptor on the host cells [28], while the NTD is involved in immune evasion and stabilization of S’s conformation [29]. S2 contains a fusion peptide (FP) and two heptad repeat regions (HR1 and HR2). The FP participates in the fusion process, while HR1 and HR2 allow for the conformational changes necessary for membrane fusion. Activation is triggered by interaction with ACE2 and the cooperation of the host cell protease involved in S cleavage, which depends on the site of fusion between the viral envelope and the plasma (through the action of TMPRSS2) or the endosomal membrane (as mediated by the host lysosomal cysteine protease cathepsin L) [30]. Therefore, S represents one of the key viral antigens, justifying the significant scientific interest in this protein throughout the pandemic [31] and in particular for vaccine development, most of which is aimed at inducing a strong immune response by eliciting the production of neutralizing antibodies (nAbs) against this protein, preventing the virus from infecting cells (Figure 4).

While vaccines induce a robust initial immune response, it has been observed that the levels of IgGs wane after approximately 73 days (52–120 days) [32], reaching levels below the protective threshold, an important fact accounted for during pandemic waves and especially regarding vulnerable individuals. This in turn necessitates the administration of booster doses to maintain protective immunity [33]. Moreover, mutations on S can impact several key features of the virus, such as its transmissibility and pathogenicity, and may prevent antigen recognition, allowing for evasion of the immune system, which effectively reduces the protective effect of vaccines (Figure 4). These mutations primarily arise spontaneously and are subject to selective pressures exerted by the host’s immune system or therapeutic interventions [34]. Additionally, concurrent infections by different variants in the same individual can lead to genetic recombination between variants, and this recombination could generate novel variants with improved fitness for transmission and survival [34].

While accounting for these mutations is crucial for vaccine development, other mutations in other genes are important as well, as they can affect immune evasion through different mechanisms. Mutations in accessory proteins like ORF3 and ORF6 disrupt innate immune responses, making these proteins key players in immune escape. Non-structural protein 1 (NSP1), for instance, significantly inhibits host antiviral defenses by interfering with the host cell’s translation machinery [35]. A specific deletion (∆500–532) in the NSP1 coding sequence has been reported to further enhance this immune evasion effect, leading to an even stronger immune escape in the variants carrying it [36].

Therefore, a periodic reassessment of vaccine measures must be performed, as the ongoing evolution of the virus dramatically reduces the effectiveness of current vaccination measures.

### 2.1. The Pre-Omicron Era

The first significant wave appeared during the summer of 2020, driven by the B.1.177 variant (Table 1 and Figure 1). It reached its peak in the fall of 2020, at which point B.1.177 had become the most prevalent strain in Europe. However, it quickly began to wane as another more concerning variant, B.1.1.7 (Alpha), emerged in the UK. Alpha was the first variant to clearly show enhanced fitness, with an estimated transmissibility increase of roughly 50% [37]. This success was largely attributable to several mutations in the S protein, which improved its affinity with the ACE2 receptor.

**Figure 5:**
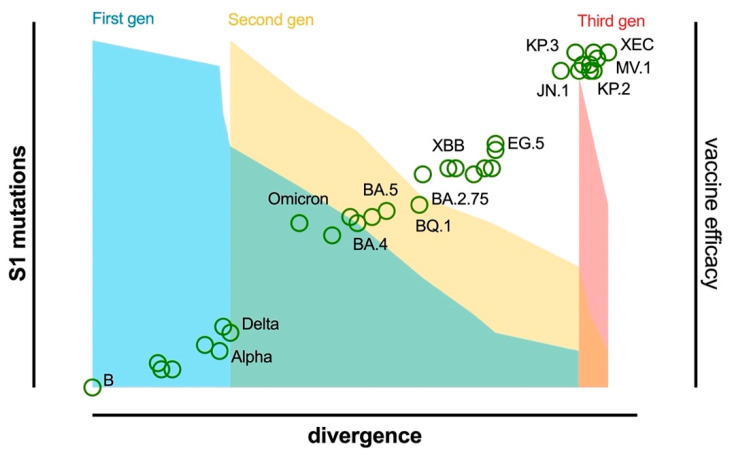
Vaccine effectiveness as a function of the viral evolution. This figure shows the relationship between SARS-CoV-2’s antigenic and genetic evolution (S1 mutations and divergence) and the estimated vaccine effectiveness (colored, shaded areas) against different viral variants (green circles). Variants are placed from left to right in order of their evolutionary distance from the original strain (measured by divergence), while each circle’s vertical position indicates the raw number of S1 protein mutations relative to the original strain. The predictions for vaccine effectiveness were inferred based on currently available neutralization and real-world vaccine performance data; they do not represent a single quantitative dataset. Data were obtained and modified from nextstrain/ncov [21] and GISAID [22] against first- (original strain (B)), second- (updated or bivalent (B/BA.1 or BA.4/5)) and third-generation (2024–2025 formula (JN.1)) vaccines.

As the pandemic was rampaging throughout the globe, the collective efforts of scientific communities produced several vaccines (Figure 1 and Figure 5), the first of which was administered in the US on the 8th of December 2020 (BNT162b2, marketed as Comirnaty). It was developed against the B.1 (Wuhan-Hu-1) strain (Figure 5). As the vaccination campaign was getting started, Alpha was spreading due to its ability to evade complete control, thanks to its superior transmission dynamic as well as its immune escape, provided by mutations such as Δ69/70 and N501Y in the RBD (Table 1 and Figure 4). All of this notwithstanding, serum neutralization studies showed that vaccines conserved their effectiveness [38] (Figure 5).

The third wave was characterized by the rise of B.1.617.2 (Delta) (Figure 2), which emerged in India in late 2020 (Table 1). Delta became infamous for its greater transmissibility and immune evasion properties, representing a significant evolutionary leap. Delta was able to spread more efficiently due to key mutations in its S protein, such as L452R, which further increased the affinity for ACE2 and conferred some resistance to nAbs. It was shown that vaccine effectiveness against infection with Delta was significantly lower than with the Alpha variant (86.7% and 98.4%, respectively) [39] (Figure 5). Delta swiftly dominated Alpha, effectively displacing it as the dominant strain by mid-2021. It spread extremely effectively among previously immunized populations with waning immunity, highlighting the need to maintain high immunity levels through booster injections and the development of updated vaccines [40]. However, the impact was particularly severe in countries with low vaccination rates.

### 2.2. The Era of Omicron and Subvariants

Up until 2021, mutations in SARS-CoV-2 variants were relatively minor. However, the evolution of the virus accelerated with the emergence of B.1.1.529/BA.1 (Omicron), which carried over 30 mutations in the S protein alone and drove the fourth wave (Table 1 and Figure 1). The rise of Omicron prompted the first major update to vaccines. In late 2022, bivalent vaccines were thus introduced, targeting both the original strain and Omicron BA.1 [41]. The year 2022 saw the rise of Omicron sublineages such as BA.4 and BA.5, which constituted the fifth wave, generating in South Africa at first but soon spreading through Europe and other regions. BA.4 and BA.5 showed an increased ability to transmit and evade host immunity to the point that sera from BA.1 or BA.2 infection or vaccination with the newly updated bivalent vaccine were no longer effective (Figure 5) [42,43,44,45,46], and breakthrough infections were routinely reported [47]. For these reasons, by the end of 2022, there was a renewed need to update the vaccines. A new bivalent booster was approved which targeted the BA.4 and BA.5 subvariants of Omicron in addition to the original strain (Figure 1 and Figure 5).

Subsequently, multiple subvariants emerged (Table 1 and Figure 2), such as BQ.1, BQ.1.1, and XBB (a hybrid strain which emerged from the genetic recombination of two distinct Omicron sublineages: BA.2.10.1 and BA.2.75). BQ.1, BQ.1.1, and XBB showed similar fitness advantages and shared S mutations which were thus convergently acquired [48,49] (Figure 3). One such mutation was R346T, which was shown to substantially reduce the effectiveness of nAbs [50]. BQ.1.1 and XBB.1 in particular showed an impressive ability to evade sera from BA.2 and BA.5 infections and were subject to only low-level neutralization from BA.5 bivalent vaccination [48,51,52,53] (Figure 5). This resulted in enhanced immune evasion of these cocirculating sublineages [54]. XBB.1 was followed by XBB.1.5 (Table 1 and Figure 2 and Figure 3) [55,56], which became a dominant strain in early 2023 thanks to its S mutation (F486P) due to increased ACE2 binding. Thankfully its pathogenicity was relatively low compared with earlier Omicron variants, despite its rapid spread [57].

Throughout 2023, new XBB.1 subvariants continued to emerge, such as XBB.1.16, which possessed several S mutations of interest, including E484L and K417N, which were known for reducing the effectiveness of nAbs. However, these expanded slowly and were no cause of particular concern [58]. Following XBB.1.16, other subvariants and recombinants appeared, such as EG.5 and FL.1.5.1 (Table 1).

EG.5 emerged in February 2023, branching from the XBB lineage (specifically from XBB.1.9.2) (Table 1 and Figure 2 and Figure 3). While not causing a surge comparable to other lineages, it still produced a noticeable uptick. EG.5 shared common mutations with its parent lineage, such as those associated with increased transmission and binding affinity, but also carried a key mutation (F456L) which was found to be associated with enhanced immune evasion. F456L and L455F are the so-called FLip mutations carried by subvariants of XBB (e.g., XBB.1.16 and XBB.2.3), and they outperformed EG.5 in neutralization tests collected from individuals who received the BA.5 bivalent booster or had breakthrough infections with BQ or early XBB sublineages (Figure 5) [59,60,61].

In July 2023, a new subvariant was identified in multiple countries, named BA.2.86. It garnered significant attention due to its unusually high number of mutations, mostly in its S protein (Table 1). The extensive mutations raised concerns about potential increases in transmissibility and immune evasion capabilities. Initial studies suggested that BA.2.86 was equipped with a greater ability to escape nAbs, given that it carried more than 25 mutations compared with XBB.1.5. However, it showed a lesser ability to escape immunity compared with previous XBB variants, particularly FLip variants and EG.5.1 [62]. Indeed, it was shown that three doses of a monovalent vaccine were ineffective against BA.2.86 [63], but the bivalent vaccinated sera could efficiently neutralize this new variant (Figure 5) [64].

In September 2023, the Food and Drug Administration (FDA) authorized a new monovalent mRNA vaccine which specifically targeted XBB.1.5 (Figure 1). Different from previous boosters, which were bivalent, this version contained only this variant, which had become the most prevalent one in the first half of 2023. Interestingly, sera from individuals vaccinated with this new monovalent vaccine showed robust efficacy against BA.2.86 as well as most XBB and FLip subvariants [65]. BA.2.86 remains a variant of interest (VOI) at the time of writing (Figure 5).

In late 2023, yet another subvariant emerged (JN.1) (Table 1), namely a descendant of the BA.2.86 (Figure 2). It is characterized by a single S mutation (L455S) which lowers the affinity for ACE2, impacting its infectivity. However, this significantly enhances its immune evasion. Due to these characteristics, JN.1 rose to dominance from late 2023 to April 2024 in the US. Up-to-date vaccines proved to still be able to elicit the production of antibodies which could still somewhat recognize JN.1, but this strain was deemed to be resistant to monovalent XBB.1.5 sera [66] and to the original monovalent vaccine [67] (Figure 5). Whereas a substantially higher titer of nAbs was identified to be elicited by bivalent vaccines (ancestral strain plus BA.4 or BA.5) [67], it is important that vaccination was still effective in preventing severe COVID-19, in part thanks to JN.1’s inability to completely evade T cell recognition [68].

By the end of the first quarter of 2024, JN.1 declined in prevalence and was replaced by newer variants derived from it (FLiRT) (Table 1 and Figure 2 and Figure 3), namely KP.2 and JN.1.16, until approximately the beginning of June, by which time KP.3 (FLuQE) and KP.3.1.1 (deFLuQE) had emerged. KP.2 contains the R346T mutation in the S1 subunit and the V1140L mutation in the S2 subunit. R346T helps compensate for the reduced affinity for the ACE2 receptor found in JN.1, while V1140L stabilizes the prefusion conformation of the S protein, which may lead to more efficient viral entry into host cells. KP.3 showed rapid spread during June 2024 but was soon displaced by the latest variant (KP.3.1.1), which appeared to be the main driver of the summer wave, along with KP.2.3 and LB.1. Though there is still limited evidence on the neutralizing capacity of vaccine-elicited antibodies against the KP.2 and KP.3 strains, preliminary evidence suggests that both possess increased immune evasion capacities compared with JN.1 [69]. The same appears to be true for the subsequent variants LB.1 and KP.2.3, which may prove to be more evasive than KP.2 and KP.3 [70].

Additionally, regarding the monovalent XBB.1.5 vaccine, individuals who received it demonstrated extremely low (50%) neutralization titer values against KP.3, LB.1, and KP.2.3 [70] (Figure 5). On the 3rd of July 2024, the European Medicines Agency (EMA) approved a monovalent JN.1 mRNA vaccine which was shown to provide a strong neutralizing response against JN.1, KP.2, KP.2.3, and LB.1 compared with the previous XBB.1.5 monovalent vaccine [71]. The FDA authorized and approved the updated version of the mRNA vaccine carrying the wording “2024–2025 formula” in late August 2024 as well as the updated peptide-based Novavax vaccine, targeting JN.1. An mRNA vaccine targeting KP.2 was approved in the US only by the end of August 2024. Currently, there are no data regarding the neutralization efficacy of KP.2 strain-targeting vaccines.

Recently, a new variant (XEC) emerged and was declared a variant under monitoring (VUM) at the end of September 2024. It is a recombination of KS.1.1 (FLiRT) and KP.3.3 (FLuQE) and likely shows an advantage thanks to its unusual T22N mutations, in combination with the FLuQE mutations. The latest variant (MV.1) first appeared in late June in India, spreading quite rapidly, and it appears to be a potential next challenger against the now-dominant DeFLuQE variants.

In accordance with the accelerating evolution of SARS-CoV-2, the frequency of release of updated vaccines has intensified in the last year. However, a Red Queen race approach to vaccine development is unsustainable. New strategies must be explored and implemented to both increase the protection offered by current vaccines, such as developing intranasal formulations which can elicit a protective mucosal IgA response, and ensure their long-term efficacy, such as through the creation of a pan-coronavirus vaccine [72].

## 3. Pan-Coronavirus Vaccine Strategies

Given the strong likelihood of the ongoing emergence of coronaviruses (CoVs) with the potential to cause disease in humans, developing a universal vaccine which offers protection across coronaviruses is of critical importance. Efforts are currently underway to develop a vaccine capable of providing broad protection against the CoV family, including SARS-CoV-2 and MERS-CoV. This would theoretically offer protection against potential future zoonotic CoVs, mitigating the possible scenario of the emergence of new pandemic variants or species.

Developing such a pan-coronavirus vaccine (PCV) is a particularly challenging feat considering the high mutation rates of RNA viruses, as well as many other issues. To date, various approaches have been proposed, including the targeting of conserved regions of the virus, such as S2, and designing vaccines which can elicit cross-reactive immune responses given the sequence homology of SARS-CoV-2 with other CoVs (between 65% and 69% in common cold CoVs such as OC43, HKU1, NL63, and 229E) [73]. While these similarities suggest the potential for developing PCV, cross-reactivity with other CoVs is mostly limited to the S protein. Thus, cross-protection may be improved by focusing on the S protein or other conserved region targets across all CoVs.

The S1 domain is more variable. In fact, RBD cross-reactivity to SARS-CoV-1 ranges from 73 to 76% and from 23 to 24% for HKU1 and OC43, respectively [73]. Given this low homology of the RBD, focusing on other targets, such as antigens which induce cross-reactive nAbs or T and B cell responses, is crucial. Cross-reactive T and B cell epitopes have been identified across the N, S2, and NSPs in ORF1 [73]. Furthermore, studies have shown that while B cell cross-reactive regions are higher in number in the early stages of infection, they tend to decline over time (3–6 months), likely due to the gradual selection of long-term memory B cells. More studies will be needed to characterize rare cross-reactive memory B cell populations. T cells have been shown to play a major role in SARS-CoV-2 protection and long-term immunity. In this context, T cell epitopes have been identified across the S protein, the highly conserved N, NSP7, and NSP13 [74].

Therefore, it may be beneficial to consider including in future vaccine designs other SARS-CoV-2 proteins which are less prone to mutations, such as ORF3, NSP3, and N. Another important factor is represented by the HLA allotypes, which restrict antigen presentation to T cells and thus may restrict the functional T cell response across CoVs [75]. In this regard, certain HLA-DRB1 have been identified with a surprisingly low number of potential epitopes in the S protein. For individuals exclusively carrying these alleles, including additional proteins in vaccines would be particularly advantageous [76]. Characterizing this phenomenon, along with the identification of epitopes of biological importance, will contribute greatly to the development of an effective PCV, as would using different approaches such as multivalent and mosaic antigen design, sequential antigen exposure, designing antigens with conserved epitopes and homotypic nanoparticle delivery, as well as nasal vaccines, which we will discuss separately.

The multivalent and mosaic antigen design approach involves combining multiple RBDs from different CoVs to create a vaccine which can elicit cross-reactive immune responses. The idea is to present a variety of antigenic targets to the immune system, increasing the likelihood of inducing broadly nAbs. Two notable examples are SpyCatcher003-mi3 nanoparticles and Quartet Nanocages. The former display several RBDs from various human and animal CoVs, eliciting a cross-reactive response, while the latter display four different RBDs, which would simplify production while still eliciting broad immunogenicity [77].

Instead of delivering all antigenic components simultaneously, sequential antigen exposure through vaccinations with different antigens could boost cross-reactive immunity more effectively than using a single mixed-antigen vaccination. Researchers from Yale University demonstrated that serial vaccinations with LNP-mRNA vaccines encoding full-length S proteins from SARS-CoV-2 Delta, SARS-CoV-1, and MERS-CoV induced stronger and more durable antibody responses in mice compared with simultaneous vaccination [78].

In addition, adeno-associated viral vector vaccines can be used to deliver designed antigens with conserved epitopes across a subset of CoVs obtained through bioinformatics analysis, an approach which has shown protection across SARS-CoV-2 variants in preclinical models [79].

Moreover, nanoparticles are designed to mimic natural viruses by presenting viral antigens in a native-like conformation on their surfaces. One prominent example is homotypic nanoparticle delivery using ferritin, a naturally occurring protein which can form nanoparticles, fused with viral antigens like SARS-CoV-2 S. S-ferritin nanoparticles, developed by the Walter Reed Army Institute of Research, present a stabilized trimeric S protein and have shown potent nAb protection against variants in preclinical studies [80]. These nanoparticles can also be combined with other vaccines to further enhance immune responses.

Finally, among the new vaccination strategies, a novel technology is being studied which mimics natural infection by combining the features of mRNA- and protein nanoparticle-based vaccines through the encoding of self-assembling enveloped virus-like particles (eVLPs) [81]. This assembly is achieved by inserting an ESCRT- and ALIX-binding region (EABR) into the S’s cytoplasmic tail, enabling the recruitment of ESCRT proteins to induce eVLP budding from cells. Recent studies on mice have demonstrated potent T cell and nAbs responses, improving neutralizing titers by more than 10 fold against Omicron-based variants for up to 3 months after a booster [81].

### Nasal Vaccines

Nasal vaccines (NV) are specifically designed to stimulate mucosal immunity (IgA), which cannot be effectively achieved by intramuscular vaccines (IMVs). The mucosal response is exceedingly important as it can prevent the virus from establishing an infection in the first site, thus significantly reducing the transmission of SARS-CoV-2. NV can also induce a systemic immune response (IgG), thus eliminating the need for an IMV and providing a more comprehensive defense against the virus. Different from IgG, anti-S IgA cannot trigger the classical complement pathway at mucosal sites, resulting in a non-inflammatory immune response. However, current vaccines generate limited amounts of both IgA and IgG in the upper and lower respiratory tracts. While IgG can passively diffuse across the epithelium to reach mucosal surfaces, the gradual decline in circulating antibodies necessitates booster vaccinations to maintain protection [82]. Mucosal vaccines can lessen viral shedding and transmission as well as prevent viral replication at the vaccination site in the event of reinfection by inducing the production of resident memory B and T cells, which can respond more quickly than systemic memory cells [83]. An additional advantage of mucosal vaccination compared with the systemic route lies in the better homing and formation of specific tissue-resident memory CD4+ and CD8+ T cells, as shown for SARS-CoV-2 [84,85,86,87,88,89,90].

Furthermore, they are easier to administer and can even be self-administered, which not only reduces the need for medical personnel but also allows for large-scale vaccination campaigns, especially in resource-limited settings such as low-income regions, as they are more thermostable and do not need cold chain logistics to be distributed. Finally, they increase compliance and reduce needle-associated adverse reactions.

NVs need to have certain features, one of which is the size of the particles. If they are too big, then they do not reach the nasal cavity, and if they are too small, then they can be inhaled into the lungs, which would raise safety concerns. Moreover, since the nasal cavity functions to efficiently clear out particles, to be effective, the particles need to remain in the nasal cavity long enough. Thus, the use of adjuvants could prove critical, and we need to find adjuvants which are effective and safe [91]. Some advancements have been made in this regard: Benetti et al. tested lipid-based nanoformulations which can deliver mRNA vaccines effectively to the nasal mucosa. Among the materials used, chitosan and lipid nanoparticles were crucial. Beyond showing resistance to lyophilization (important for storage and transport), this formulation showed effective delivery and transfection through the mRNA formulation, but preliminary in vivo studies in mice demonstrated only the induction of a local antibody response [92].

Similarly, Jakaew et al. focused on a vaccine candidate named RBD-NPs, which encapsulate the RBD of the S protein within N,N,N-trimethyl chitosan nanoparticles. With the goal of assessing the immune response of human nasal epithelial cells (HNEpCs) when exposed to RBD-NPs, they measured the production of cytokines, chemokines (e.g., IL-6, TNF-α, IFN-γ, and IL-12) and the impact of monocyte-derived dendritic cells (MoDCs) [93]. They demonstrated that this formulation provides an efficient delivery system and strongly activates both innate and adaptive immune responses in the nasal epithelium. The authors suggested that this vaccine can elicit both mucosal and systemic immune responses, as the response induced by the treatment involves the production of soluble mediators which promote the maturation of MoDCs, an interaction crucial for initiating a systemic immune response when MoDCs migrate to lymph nodes and present antigens to T cells. However, this strategy is still being studied at a preclinical level, and further research will be needed to demonstrate these preliminary findings [93].

Another preclinical study on mice made use of a chimeric protein named S2NDH, which combines two conserved regions of SARS-CoV-2: the N protein and the S2 subunit of the S protein [94]. The N fragment used is from the C-terminal domain, and the S2 fragment spans the fiber structure in the post-fusion conformation of the S protein. These regions are highly conserved among different CoVs too, which would make them ideal targets for a broad-spectrum vaccine. The formulation included Cpc oligodeoxynucleotide (ODN-39M) as a mucosal adjuvant, with which S2NDH forms spherical particles which aggregate further, potentially increasing the immunogenicity. They also tested a bivalent formulation which included the RBD of the S protein from the Delta variant. Both formulations induced humoral and cell-mediated immune responses when administered intranasally in mice. Interestingly, this included cross-reactive antibodies (IgG and IgA) against the N of different CoVs, including Omicron variants and SARS-CoV-1. The bivalent formulation showed an enhanced nAb response by targeting the RBD [94].

A different delivery strategy is being pursued by the National Institute of Health (NIH), which quite recently (July 2024) initiated a phase I clinical trial to evaluate the safety and efficacy of an experimental nasal vaccine designated MPV/S-2P, testing healthy adults aged 18–64 who previously received at least three doses of an FDA-approved or authorized mRNA COVID-19 vaccine [95]. The MPV/S-2P vaccine utilizes murine pneumonia virus (MPV) as a vector to deliver a stabilized version of the full SARS-CoV-2 S protein. MPV was chosen because it specifically targets epithelial cells in the respiratory tract and does not cause disease in humans, which is beneficial to the safety profile of this approach. Thus far, preclinical studies in non-human primates have shown the safety and efficacy of this approach. MPV/S-2P demonstrated robust systemic antibody production as well as significant local immunity in the nasal and respiratory tissues [95].

The nasal delivery route holds great promise in becoming a crucial tool in global vaccination strategies against SARS-CoV-2. However, many issues remain to be resolved, and further research is needed to fully realize its potential [91]. The advantage of mucosal vaccination compared with the systemic method can be further enhanced by combining systemic priming and intranasal boosting, leading to a stronger mucosal and systemic response than each individual route alone [82].

## 4. Hybrid Immunity and Its Long-Term Effects

Hybrid immunity develops when a subject is exposed to an antigen through both natural infection and vaccination. Because of this, it is believed that this type of immunity can lead to a more robust immune response [96]. Individuals who had COVID-19 before being vaccinated showed improvements in humoral responses both quantitatively and qualitatively compared with those who were only vaccinated [97]. They had higher titers of S-specific antibodies (though comparable to those seen in infection-naive individuals after the second dose) [98]. They also exhibited an enhancement in Fc receptor binding antibodies, particularly FcγR2a and FcγR3a [99,100].

Hybrid immunity appears to drive an immune response which targets the conserved S2 domain, which is less likely to mutate, in contrast with the vaccine-only response focusing on S1. This is also due to the fact that T cell responses are less affected by point mutations, and the polymorphism of HLA molecules in the global population makes this defense mechanism effective against variants of concern (VOCs). In fact, CD4+ and CD8+ T cells specific to the wild-type strain have been shown to exhibit cross-reactivity against the Omicron variant [101]. Indeed, despite global vaccination-induced selective pressure, the effects on S-specific CD4+ T cell responses have been marginal. This type of response could provide broader protection against new variants and be instrumental in the effort to develop a PCV strategy. Next-generation vaccines could aim to mimic the effects of hybrid immunity, particularly in targeting conserved viral regions, to provide broader and more durable protection [99]. Peptide-based vaccines are more resilient and can be stored under standard conditions, making them easily accessible. Moreover, they have the potential to elicit immune responses to specific epitopes. One of the main challenges in developing peptide-based vaccines lies in selecting the appropriate peptides. These peptides must be capable of triggering an effective immune response while avoiding excessive similarity to human proteins, which could lead to autoimmune reactions. Therefore, additional efforts must be made to identify immunogenic epitopes, possibly including both CD8+ and CD4+ T cell epitopes, which would enable a more effective and comprehensive vaccination strategy. These maps could serve as libraries which contribute to the development of vaccine platforms, which could be utilized for other viruses as well [102].

When developing a vaccine, focusing on conserved epitopes is a sensible strategy. However, a problem arises when the phenomenon known as the original antigenic sin (OAS), or the Hoskins effect, causes the immune system to focus on these epitopes at the expense of adapting to new and potentially more relevant epitopes presented by a new variant. This has been suggested as a complicating factor in vaccination strategies against SARS-CoV-2 and in part explains the rationale of why the latest iterations of the vaccine are monovalent and focus on redirecting immunity to epitopes associated with immune escape, rather than boosting immunity to conserved domains from earlier virus strains [103]. Hybrid immunity seems to modify the immune response induced by vaccines, leading to a more functionally diverse antibody profile, as they exhibit a much more diverse array of memory B cells as well as a larger number (5–10 fold more) [99]. Moreover, this appears to drive the development of T cells which are highly cross-reactive toward a broader number of variants, as well as the development of polyfunctional T cells which can produce multiple cytokines simultaneously, thus inducing a more potent immune response [104]. Both of these facts can greatly improve the immune system’s ability to respond to multiple variants, potentially including future variants.

The implications of these findings could be significant for vaccination strategies and the development of a universal PCV. Doses could be timed and designed to exploit the benefits of hybrid immunity, potentially using different vaccine platforms to enhance the breadth and depth of the immune response [105,106,107].

## 5. Implications of Main Variants and Vaccines for Pregnancy: Risks for the Mother, Fetus, and Child After Birth

Accurate assessment of the impact of COVID-19 on pregnancy requires the consideration of the variant involved in the subsequent infection, as several factors differ among them. A 2024 review highlighted that Delta posed a significant risk to mothers and children, as it carries a higher risk of causing both severe COVID-19 complications and adverse pregnancy outcomes. These data were confirmed by other studies, which showed a higher frequency of adverse outcomes during the Delta wave (such as stillbirth and preterm birth) compared with the previous period. In particular, the adjusted prevalence ratio for stillbirths was 1.55, which means that the Delta variant increased the likelihood of adverse events by 55%. Preterm births showed an adjusted prevalence ratio of 1.14 [108]. In the US, black and Hispanic women faced a disproportionately higher risk compared with white women. This disparity was mostly due to socioeconomic and environmental factors, as well as due to differences in healthcare utilization and structural racism in healthcare [109]. By decreasing the likelihood of severe outcomes (such as admissions to ICUs), vaccination has helped alleviate some of these dangers as well as flatten these disparities to some extent [110]. The risk of adverse events during the Delta wave varied depending on the stage of pregnancy; cases in the third trimester posed the greatest risk, with a 41% greater likelihood of preterm birth compared with cases occurring during the first two trimesters combined. Though to a lesser extent, cases during early gestation are still associated with an increased risk of pre-term labor and stillbirth, but this seems to be unrelated to the severity of the illness itself [108].

Regarding vertical transmission, it has been shown to be rare though quite possible event [109]. During the initial rollout of vaccinations, the absence of studies focusing on pregnancies was to be expected due to time and resource constraints, though their application to this subgroup with potential risk raised important concerns. However, historically speaking, vaccines used during pregnancy have been shown repeatedly to be safe and beneficial for both the mother and the child, with some notable examples being those for influenza and pertussis [111]. Data from multiple studies and sources then demonstrated that there were no significant adverse effects from the COVID-19 vaccines (both mRNA and adenoviral vector ones) on pregnancies or their fetuses. On the contrary, vaccination proved to be largely beneficial to both, as effective transplacental transfer of antibodies to the fetus does occur, which ultimately helps protect newborns [112].

Similarly, in cases during pregnancy, emerging evidence suggests that the timing of vaccination is relevant to neonatal protection. Indeed, some studies have shown that vaccination in the early third trimester is associated with enhanced passive immunity in newborns, as evidenced by higher IgG concentrations transferred across the placenta [112]. In terms of vaccine effectiveness, studies indicate that the administration of mRNA vaccines results in a robust maternal humoral response [111].

Ciapponi et al. conducted a comprehensive analysis on mRNA, viral vector, and inactivated virus vaccines, which included 177 studies from 41 countries. Their findings suggest that COVID-19 vaccination does not increase the risk of adverse maternal or fetal outcomes. Their results indicated consistently that there was no increased risk of miscarriage, gestational diabetes, hypertensive disorders, congenital anomalies, or preterm birth [113,114]. Some specific outcomes, like stillbirth and emergency cesarean delivery, showed statistically significant reductions associated with mRNA and viral vector vaccines. Vaccine effectiveness during pregnancy was also examined, and vaccines were shown to significantly reduce the risk of severe COVID-19 in pregnancy, particularly for mRNA vaccines, offering approximately 72% protection against severe disease across several variants, including Omicron (albeit with the lowest effectiveness). Their study also found that maternal vaccination provides protection to infants, significantly reducing the risk of hospitalization in the first 6 months of life. This effect was especially evident during the Delta wave. The incidence of adverse events following immunization was reported to be low, and the most common side effects were mild, such as pain at the injection site, fatigue, and headaches. Whereas serious adverse events were rare, no significant increase in myocarditis or seizures was observed, and the pooled proportion of anaphylaxis was extremely low [115]. One other study demonstrated that maternal vaccination protected newborn infants against SARS-CoV-2, with vaccine effectiveness (VE) reaching 64% among those neonates whose mothers received a booster dose. VE was reached in protecting neonates when given 100 days (14 weeks) or less before birth. Newborns of booster-vaccinated mothers were less likely to be born prematurely, develop respiratory distress syndrome, or spend >7 days in the NICU. Booster mRNA vaccines during pregnancy elicited a strong antibody response against the ancestral and Omicron SARS-CoV-2 strains, which were detected in umbilical cord blood [113]. Other recent studies [116,117] confirmed this evidence and support the validity and safety of the vaccine during pregnancy for the mother, the fetus, and the newborn.

## 6. Viral Evolution Impacts on Diagnosis

During the SARS-CoV-2 pandemic, virus detection in affected individuals played a crucial role. The diagnostic methods used varied based on the specific purpose. Initially, all diagnostics relied on quantitative reverse transcription polymerase chain reaction (RT-qPCR). However, the cost and processing time of this technique necessitated advancements, and these arrived in the form of loop-mediated isothermal amplification (RT-LAMP) and rapid antigen tests. The former marked a significant breakthrough in evaluating and quickly identifying potentially infectious subjects. Additionally, there arose a need to assess the immunity generated by infection and vaccination, leading to the development of serological tests to verify antibody presence and nAb tests. Finally, given the rapid mutation and evolution of this virus, sequencing tests became essential for tracking variants and updating vaccination efforts. Here, we report the features and limitations of the most used diagnostic technologies for SARS-CoV-2 detection.

### 6.1. Reverse Transcription Polymerase Chain Reaction

RT-qPCR, performed on samples primarily extracted from the upper respiratory tract (i.e., nasopharyngeal and oropharyngeal swabs), was the first reliable diagnostic method for detecting SARS-CoV-2 cases in both the early stages and throughout most of the pandemic [118,119]. This technique showed the highest accuracy in detecting the viral genome, but in the later months of 2021, the emergence of new variants caused a notable uptick in false negatives. This is attributable to mutations, the most relevant of which are in the S protein (Figure 4), such as Δ69/70, which is responsible for S gene target failure (SGTF) [6]. Consequently, the diagnostic sensitivity of PCR tests which target non-conserved genes is lowered, as unaccounted for mutations [120] prevent the successful amplification of fragments [121]. To mitigate this phenomenon, two or three genes are simultaneously amplified during the analysis. In this regard, the WHO recommends the partial amplification of several specific genes, namely the RNA-dependent RNA polymerase gene (RdRp), envelope (E), nucleocapsid (N), and membrane (M), to confirm the virus’s presence [120].

### 6.2. Reverse Transcription Loop-Mediated Isothermal Amplification

Regrettably, a mutation-induced reduction in sensitivity is not the only drawback of RT-qPCR. This technique is labor-intensive (needing more than 2 h) and requires additional reagents for RNA extraction, expensive equipment, and personnel adequately trained in molecular biology. Additionally, it requires at least two optical filters in order for probes conjugated with two or more fluorophores to be read. To mitigate some of these issues, a new diagnostic tool has been proposed, namely RT-LAMP, which is based on a colorimetric read-out [122]. RT-LAMP does not use probes; it only uses primers, and the process is faster and extremely versatile, as oligonucleotides can be obtained by several suppliers promptly and with relative ease. Another highly significant advantage is that it requires no prior RNA extraction step. Interestingly, the amplification is isothermal, meaning that it does not require temperature cycling, and rapid amplification can be obtained by adding active enzymes and specific primers [123]. This method has shown remarkable efficiency and specificity in detecting and quantifying low-abundance target sequences [124,125]. For these reasons, this test can be completed and produce a result in less than one hour. Naturally, this technique has its downsides as well. Samples require refrigeration and must be analyzed within a short time frame, and importantly, there are some concerns regarding the colorimetric read-out method, as it potentially introduces high ambiguity due to pH fluctuations, which can alter the read-out [126]. Moreover, due to the absence of thermal cycles, the risk of non-specific binding of primers increases, which increases the rate of false positives. Notwithstanding this fact, Hu et al. confirmed that RT-LAMP had higher sensitivity and specificity in diagnosing SARS-CoV-2 cases than RT-qPCR [127].

### 6.3. Rapid Antigen Detection Tests

One of the most widely used techniques involves the detection of viral antigens mainly targeting the nucleocapsid protein, which has high diagnostic value in detecting that an infection is occurring [128,129]. These tests can be carried out on samples collected in saliva or, more commonly, nasopharyngeal swabs [130]. Rapid antigen detection tests (Ag-RTDs) consist of a nitrocellulose strip coated with immobilized anti-SARS-CoV-2 gold conjugate antibodies. To serve as a control, the membrane contains anti-chicken IgY monoclonal antibodies. In essence, Ag-RDTs directly identify SARS-CoV-2 antigens by recognizing the virus’s nucleocapsid proteins via the conjugated anti-SARS-CoV-2 gold antibody on the nitrocellulose membrane [131]. Their most notable advantage is their rapidity, as they can provide results in less than one hour or, in most cases, less than 15 min [121]. Despite this rapidity, they have been widely considered accurate and have been instrumental as point-of-care testing for the management of patients during the pandemic. However, concerns have been raised due to data showing an increase in false positive rates in some challenging circumstances [132,133]. Based on this, an S-based Ag-RTD was proposed as an alternative. However, comparative studies have shown that commercial ones possess higher sensitivity [134]. Moreover, FDA reports have pointed out that antigen test performance can vary in relation to symptom onset and whether the patient is symptomatic or not, tests in asymptomatic people, in particular, are much less sensitive than it was initially reported. Nevertheless, Ag-RTDs can reliably identify patients in the course of infection, when the viral load is highest and therefore when transmissibility is greatest. Studies have been conducted which support the validity of these tests against viral evolution. One notable example is a study which involved the creation of a library of about 8000 individual amino acid substitutions in the N protein (representing more than 99.5% of all mutations). This library was then used to test 17 antibodies from 11 commercially available rapid antigen tests, which were also tested against samples from patients who had contracted different circulating variants at the time up to BA.1. The study showed that all 17 antibodies maintained their ability to recognize the mutated N proteins [135].

### 6.4. Genome Sequencing

During the early stages of the pandemic, the widespread adoption of genome sequencing technology (i.e., NGS) played a crucial role in the global response by allowing for the timely identification of many patients with COVID-19, aiding in understanding the virus, tracing the source of cases, mapping transmission pathways, and tracking the emergence of variants [119,136,137,138]. Even though this high-resolution approach has been proven to be invaluable, it suffers from several shortcomings, as it requires the employment of highly skilled technicians and the operation of expensive equipment under strict laboratory conditions. In addition, the sequencing methods are significantly time-intensive, which ultimately limits their application in various areas, including routine diagnostics [118,139]. There are alternatives though. One example is nanopore sequencing, cutting-edge technology which allows faster and more reliable real-time DNA and RNA analysis for the rapid identification of mutations associated with VOCs [121].

### 6.5. Serological Tests

Finally, serological testing represents another diagnostic tool which detects specific antibodies in blood samples. It has played a limited role in the diagnosis of COVID-19 compared with RT-PCR, essentially due to the nature and features of humoral immune response. Firstly, the response is delayed since antibodies take from several days to weeks to appear after infection. Moreover, a positive test only indicates a past case; it does not carry relevant information regarding contagiousness [140]. Lastly, serologic tests have been shown to exhibit cross-reactivity with antibodies from other CoVs, which has predictably led to false positives. However, these tests can be reliably used as surveillance tools, particularly where PCR assays are unavailable [141].

### 6.6. Future Perspectives on Diagnosis

As the demand for accurate and cheap point-of care testing increases, various novel technologies are being explored. Some notable examples are CRISPR-based, artificial intelligence (AI)-, and machine learning (ML)-based technologies.

Currently, there are three major CRISPR-Cas-based detection platforms being explored: Cas9, Cas12, and Cas13. The latter of these has the greatest potential for applications in diagnosing COVID-19, as it not only possesses trans-cleavage abilities (which greatly amplify the detectable signal) but also targets RNA. Compared with the more traditional RT-PCR, this method does not require thermal cycles, thus potentially providing a result within minutes instead of hours. Moreover, compared with RT-PCR, this method could be orders of magnitude more cost-effective. In some cases, raw material costs have been estimated to be quite low (using the SHERLOCK platform) [142]. Critically, the orthogonal activity of Cas enzymes can prove useful for multiplexed detection of several targets simultaneously, as each activated enzyme can trans-cleave a different type of reporter molecule. This would effectively enable us to detect multiple variants at the same time. However, this trans-cleavage activity can lead to off-target effects and non-specific cleavage, which could increase the rate of false positives. Two CRISPR-based technologies have made the greatest strides in the diagnosis of COVID-19: SHERLOCK and DETECTR. The former received EUA approval early in the pandemic and has been employed especially in high-complexity settings, such as hospitals and large laboratories [143]. Although they show much promise, CRISPR-based systems are still in development and face several challenges, particularly in scaling for clinical use [144].

ML and AI approaches rely on medical imaging data, specifically CT scans, to predict whether a pulmonary condition is caused by COVID-19 or not by identifying specific patterns. This technology promises to make diagnostic work faster and more accurate. However, limitations remain due to narrow datasets, low data quality, and issues with clinical integration [145]. To overcome some of these issues, new datasets have been tried, such as COVID-MAH-CT, which consists of more than 4000 CT images from 133 patients. A novel 3D deep learning model trained on this dataset showed nearly 100% accuracy in diagnosing COVID-19 according to a recent study [146].

One interesting approach to COVID-19 diagnosis is based on the detection of exosomes, which would prove advantageous as they are heavily present in most body fluids. Exosomes are implicated in the transport of various viral components from infected cells to healthy ones. In the specific case of SARS-CoV-2, specific molecules such as tetraspanin CD9 and TMPRSS2 can be found on the surface of exosomes. Their role is to help the virus enter lung cells by cleaving the fusion glycoproteins. Exosome detection methods could be applied using fluorescent dyes which label specific viral proteins or RNA present in exosomes. It has also been suggested that exosome detection could be performed with CT scan technology, as well as PET and MRI technologies [121,129,147,148].

Finally, it is noteworthy to mention that the detection of viral RNA in wastewater represents an optimal tool to track the spread and emergence of variants, filling in the data gap left by traditional diagnostic methods in monitoring a population and avoiding individual testing. These tests were able to anticipate an increase in clinical cases, enabling health authorities to act promptly [149].

## 7. Pharmacological Therapies Against SARS-CoV-2

Since the beginning of the COVID-19 pandemic, treatment strategies have rapidly evolved in response to the changing dynamics of SARS-CoV-2. Several treatments for COVID-19 have been proposed, including immunomodulatory drugs, monoclonal antibodies, and antivirals. Early therapeutic approaches focused on repurposing existing drugs, such as remdesivir, lopinavir, and dexamethasone, to mitigate the severe effects of infection [150,151]. Over time, specific interventions such as monoclonal antibodies (mAbs) and antiviral agents were developed to target viral replication and neutralize the virus. The efficacy of these approaches often remains controversial or compromised by viral evolution [152].

### 7.1. Monoclonal Antibody Treatment

The mAbs have proven to be a valuable tool in the treatment and neutralization of SARS-CoV-2 since the onset of the pandemic. Despite the demonstrated efficacy of some of these, the emergence of new variants highlights the need for further efforts in the design of novel strategies to enhance their effectiveness. In this section, we discuss some approaches aimed at optimizing mAb efficacy against emerging variants (Table 2).

Bamlanivimab (LY-CoV555) is one of the first mAbs authorized by the FDA for the treatment of the virus, but this authorization was revoked due to its reduced efficacy against new variants (Table 2) [153,154]. Although bamlanivimab has shown potent neutralizing activity against the original strain, variants like Beta and Gamma may reduce its efficacy by altering key epitopes in the S protein [155]. It was also used in combination with etesevimab (LY-CoV016, also known as JS016), which improved its efficacy [155,156]. Both mAbs specifically target the S protein.

Casirivimab (REGN10933) and imdevimab (REGN10933), collectively referred to as REGN-COV2, are two non-competing mAbs which target distinct, non-overlapping epitopes on the S protein. Preclinical studies in rodents and non-human primates demonstrated that this mAb cocktail effectively reduces viral load in both the upper and lower respiratory tracts, in addition to mitigating virus-induced pathology [157]. In phase I-III clinical trials, data from the first 275 non-hospitalized patients with SARS-CoV-2 demonstrated that REGN-COV2, administered intravenously at doses of 2400 mg or 8000 mg, is associated with a significant reduction in viral titer [158]. REGN-COV2 was officially approved in November 2020 for patients over 11 years who were at high risk of viral progression [159].

Bebtelovimab (LY-CoV1404) is a highly potent nAb which specifically targets the RBD. Compared with other authorized mAbs like bamlanivimab, casirivimab, and imdevimab, it demonstrated superior potency [160]. Its ability to neutralize variants, including Omicron, was notably higher, and it maintained its binding and neutralizing efficacy, positioning it as a strong candidate for long COVID-19 treatment [160,161]. However, due to its reduced efficacy against newer Omicron subvariants, such as BQ.1 and XBB, the FDA revoked its emergency use authorization, as bebtelovimab was no longer providing sufficient protection against these rapidly emerging variants (Table 2).

Evusheld is a combination of two long-acting mAbs (cilgavimab and tixagevimab) classified as class II and III which act on the S protein to limit its movement. It is designed to provide both prophylaxis and early treatment against illness, particularly in high-risk individuals, such as those who are immunocompromised. These mAbs target distinct epitopes on the S protein. Evusheld was authorized by the FDA in December 2021 for individuals over 12 years old [162,163]. Evidence further confirmed significant reductions in hospitalizations and mortality rates, especially in immunocompromised people, but the standard dosage was insufficient against BA.1, leading to the recommendation of an increased dosage [164]. However, new subs have shown resistance to several mAbs, including Evusheld [164,165,166], and for this reason, its authorization was revoked (Table 2).

Sotrovimab (VIR-7831) in clinical phase III trials reduced the risk of hospitalization or death by 85% compared with a placebo in high-risk adults with COVID-19. Sotrovimab demonstrated its neutralization capacity against Omicron, but this efficacy was reduced (Table 2) [167].

Regdanvimab (CT-P59) is an effective mAb, blocking the interaction between the RBD and ACE2. In animal models, regdanvimab reduced viral load and alleviated the symptoms of infection [168]. Clinical trials showed a reduction in hospitalization and oxygen therapy needs among patients receiving regdanvimab, particularly in high-risk groups [169]. Studies also showed its neutralizing activity against many variants, though the efficacy was somewhat reduced compared with that against the wild-type virus [170]. Regdanvimab received approval in South Korea in September 2021 for use in patients over 50 years old with underlying conditions, as well as patients with moderate COVID-19 symptoms. It was also granted marketing authorization by the EMA for patients at risk of progressing to severe COVID-19 [171].

Pemivibart (Pemgarda) was approved by the FDA in May 2024, and it is currently under review by the EMA (Table 2). Pemivibart is specifically designed for pre-exposure prophylaxis, providing protection to immunocompromised patients who cannot generate a strong enough immune response through vaccination [172].

All clinically authorized mAbs were inactive against new subvariants [173]. Omicron has been shown to evade over 85% of 247 human mAbs. XBB.1.9.3, XBB.1.5, XBB.2.9, and BQ.1.1.45 have shown a marked reduction in susceptibility to neutralization by many of the mAbs currently available, and they exhibit the highest levels of antibody evasion seen in any variants [174,175,176].

The resistance of BQ.1.1 and XBB.1 is attributed to specific mutations such as R346T, K444T, N460K, and F486S, which hinder the binding of antibodies that target these regions of the S protein [60,175,177]. For example, B.1.1.529 carries 15 mutations in the RBD and over 30 mutations in total, impacting nAbs which target both the RBD and NTD (Figure 4) [167]. Despite the significant changes in antibody resistance, the receptor-binding affinity to ACE2 of BQ.1 and XBB remains similar to their predecessors, suggesting that antibody evasion, rather than increased ACE2 binding, is driving their rapid spread [60].

Variants with key mutations like E484K and K417N significantly reduce the efficacy of mAbs such as bamlanivimab and casirivimab by altering the S protein’s structure, making it harder to bind effectively and neutralize the virus. While this variant shows partial resistance to mAbs targeting the NTD, it remains largely susceptible to most mAbs recognizing the RBD [154]. Moreover, sotrovimab and bebtelovimab remain effective against Omicron mutations and have shown a substantial reduction in hospitalizations and mortality in clinical trials, but in the case of bebtelovimab, we do not have sufficient evidence to confirm the actual efficacy of these treatments compared with other mAbs [178,179,180]. In particular, most mAbs showed a dramatic reduction in their neutralizing ability. For example, some treatments exhibited over a million-fold reduction in efficacy against several Omicron variants [176].

The rise of variants like Delta and Omicron has introduced new challenges, with many therapies showing reduced efficacy against these mutated forms of the virus. There is a need for next-generation mAbs which can target multiple epitopes to overcome the resistance posed by such mutations, particularly for the treatment of immunocompromised patients [60,67,154,173,176,177].

**Table 2:** Current approved monoclonal antibodies against SARS-CoV-2 and efficacy against variants.

Monoclonal Antibody	Variants Efficacy	Status	FDA Setting	Clinical Trial
Bamlanivimab (LY-CoV555)	Not effective against variants.	Authorization revoked	Adults and pediatric patients (12 years of age and older and weighing at least 40 kg) with positive results	Cohort: 1097 people [181]
REGN-COV2 (Casirivimab (REGN10933) and Imdevimab (REGN10933))	Not effective against variants.	FDA partially revoked, EMA-approved	Adults and pediatric individuals (12 years of age and older and weighing at least 40 kg) who were at high risk for progression to severe COVID-19	Cohort: 799 people [159]
Bebtelovimab	Effective against variants, not Omicron BQ.1 or XBB.	Authorization revoked	Adults and pediatric patients (12 years of age and older and weighing at least 40 kg) with positive results	Cohort: 706 people [182]
Evusheld (Cilgavimab + Tixagevimab)	Effective against variants, not Omicron.	FDA revoked, EMA-approved	Adults and pediatric patients (12 years of age and older and weighing at least 40 kg) who were immunocompromised	Cohort: 5197 people [183]
Sotrovimab (VIR-7831) (Xevudy)	Effective against variants. Efficacy reduced in Omicron.	FDA revoked, EMA-approved	Adults and pediatric patients (12 years of age and older and weighing at least 40 kg) with positive results; not recommended for those requiring oxygen	Cohort: 583 people [184]
Regdanvimab (CT-P59) (Regkirona)	Proven efficacy. Efficacy reduced in new variants.	EMA-approved		Cohort: 1315 people [185]
Pemgarda (Pemivibart)	Efficacy against new Variants.	FDA-approved, EMA in review	Adults and pediatric patients (12 years of age and older and weighing at least 40 kg) who were immunocompromised	Cohort: 775 people [186]

### 7.2. Antiviral Treatment

As alluded to above, since the insurgence of the COVID-19 pandemic, host immune pressure derived from either a previous case or vaccination has promoted the selection and spread of novel variants partially escaping immune recognition via vaccines and neutralization by mAbs. However, while mAbs and vaccine-induced nAbs mainly target the highly flexible S protein [187,188,189,190], the three FDA-approved antivirals target alternative viral proteins and maintain high activity versus novel variants [151,191,192,193]. During the extremely early stages of the pandemic, several broad-spectrum antivirals were investigated through extensive drug-repurposing approaches (Table 3) [194]. Drugs from different origins and with heterogeneous mechanisms of action were evaluated, including several host-targeting compounds such as antimalarial hydroxychloroquine, which targets SARS-CoV-2 entry [195,196] and the antiparasitic drug Ivermectin, which has been shown to inhibit replication of several viruses, likely due to its ability to interfere with nuclear import mediated by the importin alpha/beta pathway [197,198,199,200] as well as previously characterized antivirals, including antiretrovirals [201], and anti-influenza drugs [202]. Despite encouraging results in vitro, only remdesivir, molnupiravir, and nirmatrelvir were approved for clinical use (Table 3) [203]. Indeed, neither hydroxyquinoline nor ivermectin have been shown to be effective in any way when evaluated in controlled clinical trials. Since such enzymes are essential for viral replication but are not the target of strong humoral responses, they are highly conserved across the variants. Indeed, on the one hand, mutations mediating drug resistance would result in decreased viral fitness, and on the other hand, they would not confer any evolutionary advantage in terms of virus spread across a population [193], making them attractive targets even for future variants [204].

#### 7.2.1. RNA-Dependent RNA Polymerase Inhibitors

RdRp is a highly conserved enzyme essential for the transcription and replication of the SARS-CoV-2 genome and is considered an effective target for COVID-19 treatment [144,205]. The RdRp structure comprises NSP12 and two accessory non-structural proteins: NSP7 and NSP8. The active site within the N-terminal nidovirusRdRp-associated nucleotidyltransferase domain (NiRAN) of NSP12 can be blocked by small nucleoside analogues [205]. The incorporation of these nucleoside analogues can either lead to the inhibition of RNA synthesis or the lethal accumulation of mispaired nucleobases [205,206]. RdRp inhibitors, such as favipiravir, remdesivir, ribavirin, and galidesivir, have been a high priority since the beginning of COVID-19 trials. However, SARS-CoV-2 possesses a nonstructural protein, NSP14, with amino-terminal domain coding for a proofreading exonuclease (ExoN). The ExoN is capable of excising incorporated nucleoside analogs by virtue of its 3′–5′ exonuclease proofreading activity. This is believed to compromise the action of most nucleosides and nucleotides analogous to varying extents, depending on the type of nucleoside analog chemistry.

Remdesivir (RDV; Veklury, GS-5734) (Table 3) is an adenosine nucleoside analogue originally identified as a potent inhibitor of several RNA viruses, such as yellow fever virus (YFV), Dengue virus type 2 (DENV-2), influenza A, parainfluenza 3, and SARS. Given its efficacy in vitro and in animal models against EBOV, it was clinically evaluated during the EBOV outbreak in 2014, only to be proven to be poorly efficient in preventing mortality, although fairly safe [207]. Given its inhibitory activity against SARS-CoV-1 and MERS and its safety profile, it was among the first drugs clinically evaluated against SARS-CoV-2. RDV is a monophosphoramidate prodrug which, after cell entry, is metabolized into its active form: RDV-triphosphate (RDV-TP, GS443902). The incorporation of RDV-TP into nascent RNA instead of ATP causes delayed chain termination due to the interaction between the RDV-monophosphate 1′-cyano with S861 in the RdRp, inhibiting further translocations of the enzyme, with the addition of three more nucleotides before RNA synthesis stalls [208,209]. Furthermore, RDV can bypass the exoribonuclease proofreading activity of CoVs inhibiting viral RNA synthesis [210,211].

Clinical trials such as ACTT-1 (NCT04280705) and PINETREE (NCT04501952) have demonstrated the ability of intravenous injections of RDV to slightly but significantly reduce recovery by 5 days time for hospitalized patients with lower respiratory tract infections and severe symptoms compared with a placebo (median of 10 days, compared with 15 days with placebo) [151,192,205,206]. As a result of such investigations, in May 2020, RDV was the first drug to receive FDA emergency approval for use in adult and pediatric patients 12 years of age and older who tested positive for SARS-CoV-2 and required hospitalization [212]. Since the initial outbreak of COVID-19, several waves of VOCs have emerged. Despite the rapid spread of variants, naturally occurring alterations to the RdRp have been scarce. Even though mutations such as P323L were shown to increase replication, changes to the sequence of viral polymerase are not generally favored and lead to a reduction in replication fitness [213,214]. The effect of RDV pressure was reported to cause non-synonymous mutations, such as S759A and V792I in nsp12, for the viral RdRp. These mutations were shown to cause resistance to RDV by reducing the preference for RDV-TP and the concentration of UTP required for template-dependent inhibition. However, only the S759A substitution was detected in clinical strains, and the lineage bearing this mutation showed considerably lower replication capabilities [214]. Recently, it has been reported that prolonged SARS-CoV-2 cases in immunocompromised patients resulted in the selection of naturally occurring viral strains bearing the V792I mutation, with similar transmissibility to the wild-type virus in a golden Syrian hamster model despite a brief delay in the detection of the virus, implying no impact on the transmission or replication rate of the virus [215]. Despite the aforementioned rare mutations, the RdRp has generally remained conserved among the variants, and the antiviral compounds targeting this enzyme have maintained their effectiveness [191].

However, it must be highlighted that the clinical efficacy of RDV has been severely limited by the need to be administered quite early in cases to be effective and by its route of administration, requiring intravenous injection for three consecutive days [155].

Molnupiravir (MLP, Lagevrio) (Table 3) is an oral form of the β-dN4-hydroxycytidine (EIDD-1931, NHC) prodrug. This compound had previously shown an inhibitory effect on RNA viruses such as Venezuelan equine encephalitis virus, influenza A and B, EBOV, SARS, and MERS, and thus it was promptly tested against SARS-CoV-2 [216,217,218].

Following the administration and systemic circulation, NHC host kinases facilitate its conversion into its active form: NHC triphosphate. After activation, MLP competes with natural CTP or UTP to be incorporated into the viral template strand. As a result, NHC triphosphate misleads RNA polymerase to incorporate either guanosine or adenosine, causing the accumulation of mispaired nucleobases through an increase in G-to-A and C-to-U transition errors, a mechanism eventually leading to the inability of virus replication through error catastrophe [219,220].

In vitro studies evaluating the 50% inhibitory concentration (IC_50_) of MLP in various cell lines, including Vero E6, Calu-3, and human tracheal and small airway epithelial cells, showed a dose-dependent inhibition of SARS-CoV-2 replication [151,221]. The efficacy of MLP against SARS-CoV-2 in patients was demonstrated by MOVe-OUT phase III clinical trials, in which the administration of MLP reduced the risk of hospitalization and death in cases with pre-Omicron variants. Based on such evidence, in November 2021, MLP was granted by the FDA emergency use authorization for patients 18 years of age and older with mild-to-moderate COVID-19 and who were at risk of disease progression, hospitalization, or death [222].

The peculiar mechanism of action of MLP results in an extremely highly genetic barrier [151], and the drug remains effective against all SARS-CoV-2 variants of concern, including RDV-resistant strains [151,193,223]. However, due to the activity of MLP as a mutagenic ribonucleoside analogue, its use needs to be strictly limited. Indeed, MLP administration for longer than 5 days in immunocompromised patients has been suggested to promote the selection of novel viral variants [223,224]. For this reason, and to prevent the possibility of teratogenesis and fetal toxicity in pregnant women, it can only be administered for five consecutive days [225,226]. Furthermore, a platform-adapted, randomized controlled trial, PANORAMIC, demonstrated minimal effectiveness for MLP in reducing hospitalization of high-risk outpatients with incomplete vaccination during the Omicron era [227]. On 21 June 2023, Merck withdrew its application for marketing authorization of MLP in Europe for the treatment of COVID-19 in adults. This occurred after the EMA recommended refusing marketing authorization based on evaluation of data provided by the company, with which MLP’s effectiveness could not be clearly and conclusively demonstrated [228,229].

#### 7.2.2. Protease Inhibitors

The SARS-CoV-2 main protease (Mpro) rapidly emerged as an alternative attractive target for antiviral therapy, being responsible for the maturation of viral polyproteins pp1a and pp1ab [230]. The catalytic site of each subunit of Mpro homodimer contains a dyad composed of a cysteine at position 145 (Cys145) [231]. Neutralization of this site makes the enzyme inactive. This can be achieved by using small molecules which covalently or non-covalently occupy this space.

Nirmatrelvir (NMV, PF-07321332) (Table 3) is the most successful of such molecules. Originally under development against SARS-CoV, the structure of NMV consists of a nitrile as the warhead, a canonical γ-lactam, a bicyclic proline derivative, a tert-leucine structure, and a trifluoroacetamide group [231]. NMV utilizes its warhead to bind to the catalytic site of the Mpro covalently, neutralizing its activity [232].

The inhibitory activity of NMV was demonstrated against SARS-CoV-2 variants in various cell lines, including Vero E6, Calu-3, and primary human airway organoids [193,232]. Moreover, clinical trials such as EPIC-HR have depicted the efficacy of treatment with NMV [233]. In these trials, NMV was co-administered with ritonavir to prevent its premature metabolism by liver cytochrome P450 3A4 (CYP3A4), thus boosting its therapeutic concentration [234]. EPIC-HR trials have demonstrated that administration of nirmatrelvir plus ritonavir was strongly effective as an early treatment for patients with moderate COVID-19 [233]. Based on this study, nirmatrelvir/ritonavir (Paxlovid) was approved in December 2021 for adult patients with mild-to-moderate COVID-19 and who were at risk of disease progression.

Currently, Paxlovid is the antiviral drug used the most for COVID-19 treatment. Given its widespread use, and considering the high mutation rate of SARS-CoV-2, the emergence of NMV-resistant variants is not far off. Several missense point mutations have been identified which influence the catalytic activity of Mpro across different variants. The mutations are G15S, which is most prevalent in the Lambda variant, K90R, which is most prevalent in the Beta variant, and P132H, which is most prevalent in the Omicron variant. No missense mutation in the Mpro of the Delta variant was identified compared with the wild type [232]. These mutations do not introduce any major alterations in the structure of the enzyme, especially in the active site. For this reason, the catalytic activity of Mpro and its sensitivity to protease inhibitors are likely to remain. Furthermore, studies have suggested several possible pathways of NMV resistance [204]. These pathways were described as the accumulation of mutations in a stepwise manner, specifically mutations such as T21I, P252L, and T304I, which were considered precursor mutations, followed by E166V, L50F, and S144A. The aforementioned single-point mutations cause low levels of resistance but significantly reduce the replication fitness of viruses. Only double mutations of E166V and T21I or L50F maintain the replication capabilities of the WT along with NMV resistance [204,215]. Furthermore, naturally occurring mutations were reported to have emerged in immunocompromised cases with long COVID, such as T169I in the nsp5 protease. These mutations were detected to promote low-level resistance to NMV, but compared with the parental strains, no significant alteration regarding their transmissibility was observed in golden Syrian hamsters. Contact animals were successfully infected in the presence of infected hamsters and showed a high viral load on day 14 like the infected animals, raising potential concerns [215]. The administration of Paxlovid requires some considerations, such as possible drug–drug interactions with concomitant medications or the effects of the drug on patients with severe renal issues or hypersensitivity reactions. Furthermore, studies have shown cases of COVID-19 rebound after the completion of treatment [235]. In summary, Paxlovid was shown to remain effective against all SARS-CoV-2 variants reported thus far, but due to the constant emergence of new variants and the complications mentioned above, the activity of this drug should be monitored and evaluated in future studies.

**Table 3:** Current approved antivirals for SARS-CoV-2 variants.

Antiviral	Status	FDA Setting	Clinical Trial
Remdesivir (Veklury)	FDA- and EMA-approved	Adult and pediatric patients 12 years of age and older requiring hospitalization and weighing at least 40 kg.	Cohort: 1062 people [205]
Molnupiravir (Lagevrio)	FDA authorization and EMA withdrawal	Adults with positive results for direct SARS-CoV-2 viral testing who are at high risk of progression to severe COVID-19 and for whom alternative COVID-19 treatment options authorized by the FDA are not accessible or clinically appropriate.	Cohort: 1433 people [222]
Nirmatrelvir/ritonavir (Paxlovid)	FDA- and EMA-approved	Mild-to-moderate COVID-19 in adults who are at high risk for progression to severe COVID-19, including hospitalization or death.	Cohort: 2246 people [233]

## 8. Conclusions

SARS-CoV-2’s ongoing evolution presents significant challenges for global public health, diagnostics, treatment, and vaccine development. Continued investment in genomic surveillance, adaptive diagnostic strategies, and next-generation vaccine technologies will be crucial for managing the long-term impacts of this evolving virus. Future research should focus on elucidating the complex interplay between viral evolution, host immunity, and environmental factors to inform more effective and durable interventions. As we move forward, a multidisciplinary approach integrating virology, immunology, epidemiology, and data science will be crucial for anticipating and mitigating the impacts of SARS-CoV-2’s evolution. The global nature of the pandemic underscores the need for coordinated international efforts and equitable access to resources to effectively combat this ongoing threat to public health.

## Figures and Tables

**Table 1:** Summary table of SARS-CoV-2 variants with their WHO classifications and key genetic features.

Variant	PANGO Lineage	Date of Designation	Risk Assessment Update	Designation	Next Strain Clade	Relevant Genetic Features	Earliest Documented Samples	Prototype GenBank Accession Number
Alpha	B.1.1.7	18 Dec 2020	20 Sep 2021	Previous VOC	20I (V1)	S:N501Y S:Δ69/70 S:P681H S:T716I S:S982A	United Kingdom, Sep 2020	MZ344997.1
Beta	B.1.351	18 Dec 2020	20 Sep 2021	Previous VOC	20H (V2)	S:E484K	South Africa, May 2020	MW598419.1
Gamma	P.1	11 Jan 2021	20 Sep 2021	Previous VOC	20J (V3)	S:E484K	Brazil, Nov 2020	MW642250.1
Epsilon	B.1.427/B.1.429	5 Mar 2021	6 Jul 2021	VOI	21C	S:L452R	USA, Mar 2020	MW453103.1
Zeta	P.2	17 Mar 2021	6 Jul 2021	VOI	20B/S.484K	S:E484K	Brazil, Apr 2020	MW523796.1
Eta	B.1.525	17 Mar 2021	20 Sep 2021	VOI	21D	S:E484K S:F888	Multiple countries, Dec 2020	MW560924.1
Theta	P.3	24 Mar 2021	6 Jul 2021	VOI	21E	S:E484K S:N501Y	Philippines, Jan 2021	NA
Iota	B.1.526	24 Mar 2021	20 Sep 2021	VOI	21F	S:E484K S:D614G	USA, Nov 2020	MW643362.1
Kappa	B.1.617.1	4 Apr 2021	20 Sep 2021	VOI	21B	S:L452R S:E484Q	India, Oct 2020	MW966601.1
Delta	B.1.617.2	11 May 2021	Previous VOC: 7 Jun 2022	Previous VOC	21A, 21I, 21J	S:L452R S:T478K S:P681R S:D614G	India, Oct 2020	MZ009823.1
Lambda	C.37	14 Jun 2021	9 Mar 2022	VOI	21G	S:L452Q S:F490S	Peru, Dec 2020	MW850639.1
Mu	B.1.621	30 Aug 2021	9 Mar 2022	VOI	21H	S:T95I S:Y144S S:R346K S:E484K S:N501Y	Colombia, Jan 2021	OQ248293.1
Omicron	B.1.1.529 (includes BA.1, BA.2, BA.3, BA.4, BA.5)	26 Nov 2021	-	VOC	21K, 21L, 21M, 22A, 22B, 22C, 22D	S:R346K S:L452X S:F486V	Multiple countries, Nov 2021	OL672836.1
BA.2.75	BA.2.75	06 Jul 2022	10 Apr 2024	VUM	22D	BA.2 + S:K147E S:W152R S:F157L S:I210V S:G257S S:D339H S:G446S S:N460K S:Q493R reversion	31 Dec 2021	ON990685.1
BQ.1	BQ.1	21 Sep 2022		VUM	22E	BA.5 + S:R346T S:K444T S:N460K	07 Feb 2022	OP412163.1
XBB	XBB	12 Oct 2022	10 Apr 2024	VUM	22F	BA.2+ S:V83A S:Y144- S:H146Q S:Q183E S:V213E S:G252V S:G339H S:R346T S:L368I S:V445P S:G446S S:N460K S:F486S S:F490S	19 Aug 2022	OR098785.1
BA.5	BA.5	20 Nov 2022		VUM	22B, 22E	BA.5 + one or more of these mutations: S:R346X S:K444X S:V445X S:N450D or S:N460X	07 Feb 2022	ON249995.1
BA.4.6	BA.4.6	20 Nov 2022		VUM	22A	BA.4 + S:R346T S:N658S	20 Jul 2020	OR325409.1
BA.2.3.20	BA.2.3.20	20 Nov 2022		VUM	21L	BA.2 + S:M153T S:N164K S:H245N S:G257D S:K444R S:N450D S:L452M S:N460K S:E484R	15 Aug 2022	PP847689.1
XBB.1.5	XBB.1.5	11 Jan 2023	7 Jun 2024	VOI	23A	Recombinant of BA.2.10.1 and BA.2.75 sublineages, i.e., BJ.1 and BM.1.1.1, with a breakpoint in S1. XBB.1 + S:F486P (similar Spike genetic profile as XBB.1.9.1) Includes XBB.1.5.70 (23G): XBB.1.5 + S:L455F and S:F456L	21 Oct 2022	OP790748.1
CH.1.1	CH.1.1	8 Feb 2023		VUM	22D	BA.2.75 + S:L452R S:F486S	27 Jul 2022	PP848047.1
XBF	XBF	8 Feb 2023		VUM		Recombinant of BA.5.2.3 and CJ.1 (BA.2.75.3 sublineage) BA.5 + S:K147E S:W152R S:F157L S:I210V S:G257S S:G339H S:R346T S:G446S S:N460K S:F486P S:F490S	27 Jul 2022	PP848029.1
BF.7	BF.7	9 Feb 2023		VUM	22B	BA.5 + S:R346T	24 Jan 2022	PP848045.1
XBB.1.9.1	XBB.1.9.1	30 Mar 2023	10 Apr 2024	VUM	23D	Recombinant of BA.2.10.1 and BA.2.75 sublineages (i.e., BJ.1 and BM.1.1.1 XBB.1 + S:F486P S:Q613H)	05 Dec 2022	PP846633.1
XBB.1.16	XBB.1.16	17 Apr 2023	7 Jun 2024	VOI	23B	Recombinant of BA.2.10.1 and BA.2.75 sublineages (i.e., BJ.1 and BM.1.1.1 XBB.1 + S:E180V S:K478R S:F486P)	09 Jan 2023	PP846659.1
XBB.1.9.2	XBB.1.9.2	26 Apr 2023	10 Apr 2024	VUM	23D	XBB.1 + S:F486P	05 Dec 2022	PP846644.1
XBB.2.3	XBB.2.3	17 May 2023	10 Apr 2024	VUM	23E	Recombinant of BA.2.10.1 and BA.2.75 sublineages (i.e., BJ.1 and BM.1.1.1 XBB + S:D253G S:F486P S:P521S)	09 Dec 2022	PP846522.1
EG.5	EG.5	09 Aug 2023	28 Jun 2024	VOI	Not Assigned	XBB.1.9.2 + S:F456L, which includes EG.5.1 (23F): EG.5 + S:Q52H HK.3 (23H): EG.5 + S:Q52H S:L455F HV.1: EG.5 + S:Q52H S:F157L S:L452R	17 Feb 2023	OQ873579.1
DV.7	DV.7	23 Oct 2023	10 Apr 2024	VUM	23C	CH.1.1 + S:N185D S:L858I	19 Jan 2023	PP846399.1
BA.2.86	BA.2.86	21 Nov 2023	-	VOI	23I	Mutations relative to BA.2	24 Jul 2023	PP092736.1
JN.1	JN.1	18 Dec 2023	Updated on 15 Apr 2024	VOI	24A	BA.2.86 + S:L455S	25 Aug 2023	PP846619.1
JN.1.7	JN.1.7	03 May 2024	-	VUM	24A	JN.1 + S:T572I S:E1150D	25 Sep 2023	NA
JN.1.18	JN.1.18	03 May 2024	-	VUM	24A	JN.1 + S:R346T	11 Nov 2023	NA
KP.2	KP.2	03 May 2024	-	VUM	24B	JN.1 + S:R346T S:F456L S:V1104L	02 Jan 2024	NA
KP.3	KP.3	03 May 2024	-	VUM	24C	JN.1 + S:F456L S:Q493E S:V1104L	11 Feb 2024	NA
LB.1	LB.1	28 Jun 2024	-	VUM	24A	JN.1 + S:S31- S:Q183H S:R346T S:F456L	26 Feb 2024	NA
KP.3.1.1	KP.3.1.1	19 Jul 2024	-	VUM	24E	KP.3 + S:S31-	27 Mar 2024	NA
XEC	XEC	24 Sep 2024	-	VUM	24F	JN.1 + S:T22N S:F59S S:F456L S:Q493E S:V1104L	16 May 2024	NA

VOI = variant of interest; VOC = variant of concern; VUM = variant under monitoring; NA = not available.

## References

[1] WHO 20 January 2020 Novel Coronavirus (2019 n-CoV) Situation Report-1. https://www.who.int/docs/default-source/coronaviruse/situation-reports/20200121-sitrep-1-2019-ncov.pdf.

[2] WHO 22 December 2020. Weekly Epidemiological Update. https://www.who.int/publications/m/item/weekly-epidemiological-update---22-december-2020#:~:text=Globally%20in%20the%20past%20week,the%20start%20of%20the%20pandemic..

[3] WHO 28 December 2021. Weekly Epidemiological Update on COVID-19. Edition 72. https://iris.who.int/handle/10665/350973.

[4] Angius F., Pala G., Manzin A. (2021). SARS-CoV-2 and Its Variants: The Pandemic of Unvaccinated. Front. Microbiol..

[5] Giovanetti M., Benedetti F., Campisi G., Ciccozzi A., Fabris S., Ceccarelli G., Tambone V., Caruso A., Angeletti S., Zella D. (2021). Evolution patterns of SARS-CoV-2: Snapshot on its genome variants. Biochem. Biophys. Res. Commun..

[6] Aleem A., Akbar Samad A.B., Vaqar S. (2024). Emerging Variants of SARS-CoV-2 and Novel Therapeutics Against Coronavirus (COVID-19). StatPearls.

[7] WHO Coronavirus (COVID-19) Situation Reports. https://www.who.int/emergencies/diseases/novel-coronavirus-2019/situation-reports.

[8] WHO 21 December 2022. Weekly Epidemiological Update on COVID-19. Edition 123. https://iris.who.int/handle/10665/365535.

[9] Willyard C. (2022). What the Omicron wave is revealing about human immunity. Nature.

[10] Pather S., Madhi S.A., Cowling B.J., Moss P., Kamil J.P., Ciesek S., Muik A., Tureci O. (2023). SARS-CoV-2 Omicron variants: Burden of disease, impact on vaccine effectiveness and need for variant-adapted vaccines. Front. Immunol..

[11] Sreepadmanabh M., Sahu A.K., Chande A. (2020). COVID-19: Advances in diagnostic tools, treatment strategies, and vaccine development. J. Biosci..

[12] Dryden-Peterson S., Kim A., Kim A.Y., Caniglia E.C., Lennes I.T., Patel R., Gainer L., Dutton L., Donahue E., Gandhi R.T. (2023). Nirmatrelvir Plus Ritonavir for Early COVID-19 in a Large U.S. Health System: A Population-Based Cohort Study. Ann. Intern. Med..

[13] CDC 22 November 2022. CDC Reports on Bivalent COVID-19 Vaccine, Paxlovid Effectiveness. https://www.aha.org/news/headline/2022-11-22-cdc-reports-bivalent-covid-19-vaccine-paxlovid-effectiveness.

[14] Hansen K., Makkar S.R., Sahner D., Fessel J., Hotaling N., Sidky H. (2023). Paxlovid (nirmatrelvir/ritonavir) effectiveness against hospitalization and death in N3C: A target trial emulation study. medRxiv.

[15] Paxlovid Emergency Use Authorization. https://labeling.pfizer.com/ShowLabeling.aspx?id=17109.

[16] WHO 22 December 2023. COVID-19 Epidemiological Update. Edition 162. https://iris.who.int/handle/10665/375379.

[17] Alqahtani M., Abdulrahman A., Mustafa F., Alawadhi A.I., Alalawi B., Mallah S.I. (2021). Evaluation of Rapid Antigen Tests Using Nasal Samples to Diagnose SARS-CoV-2 in Symptomatic Patients. Front. Public Health.

[18] Anand A., Vialard F., Esmail A., Ahmad Khan F., O’Byrne P., Routy J.P., Dheda K., Pant Pai N. (2024). Self-tests for COVID-19: What is the evidence? A living systematic review and meta-analysis (2020–2023). PLoS Glob. Public Health.

[19] Mukoka M., Sibanda E., Watadzaushe C., Kumwenda M., Abok F., Corbett E.L., Ivanova E., Choko A.T. (2023). COVID-19 self-testing using antigen rapid diagnostic tests: Feasibility evaluation among health-care workers and general population in Malawi. PLoS ONE.

[20] News U.T. COVID Variant XEC Sees Rapid Global Growth: What to Know About the New Strain. https://www.usatoday.com/story/news/health/2024/09/16/xec-covid-variant/75253344007/.

[21] Hadfield J., Megill C., Bell S.M., Huddleston J., Potter B., Callender C., Sagulenko P., Bedford T., Neher R.A. (2018). Nextstrain: Real-time tracking of pathogen evolution. Bioinformatics.

[22] Elbe S., Buckland-Merrett G. (2017). Data, disease and diplomacy: GISAID’s innovative contribution to global health. Glob. Chall..

[23] Mike-Honey COVID-19-Genomes. https://github.com/Mike-Honey/covid-19-genomes?tab=readme-ov-file.

[24] Cook T.M., Farrar J.J. (2021). COVID-19 vaccines: One step towards the beginning of the end of the global impact of the pandemic. Anaesthesia.

[25] Khan K., Lustig G., Romer C., Reedoy K., Jule Z., Karim F., Ganga Y., Bernstein M., Baig Z., Jackson L. (2023). Evolution and neutralization escape of the SARS-CoV-2 BA.2.86 subvariant. Nat. Commun..

[26] Wee L.E., Sim X.Y.J., Conceicao E.P., Aung M.K., Goh J.Q., Yeo D.W.T., Gan W.H., Chua Y.Y., Wijaya L., Tan T.T. (2020). Containment of COVID-19 cases among healthcare workers: The role of surveillance, early detection, and outbreak management. Infect. Control. Hosp. Epidemiol..

[27] Jeyanathan M., Afkhami S., Smaill F., Miller M.S., Lichty B.D., Xing Z. (2020). Immunological considerations for COVID-19 vaccine strategies. Nat. Rev. Immunol..

[28] Winger A., Caspari T. (2021). The Spike of Concern-The Novel Variants of SARS-CoV-2. Viruses.

[29] Hamming I., Timens W., Bulthuis M.L., Lely A.T., Navis G., van Goor H. (2004). Tissue distribution of ACE2 protein, the functional receptor for SARS coronavirus. A first step in understanding SARS pathogenesis. J. Pathol..

[30] Peng R., Wu L.A., Wang Q., Qi J., Gao G.F. (2021). Cell entry by SARS-CoV-2. Trends Biochem. Sci..

[31] Hamed S.M., Sakr M.M., El-Housseiny G.S., Wasfi R., Aboshanab K.M. (2023). State of the art in epitope mapping and opportunities in COVID-19. Future Sci. OA.

[32] Ibarrondo F.J., Fulcher J.A., Goodman-Meza D., Elliott J., Hofmann C., Hausner M.A., Ferbas K.G., Tobin N.H., Aldrovandi G.M., Yang O.O. (2020). Rapid Decay of Anti-SARS-CoV-2 Antibodies in Persons with Mild COVID-19. N. Engl. J. Med..

[33] Zhang B., Huo J., Huang Y., Teo S.C., Li Y.F., Toh L.K., Lam K.P., Xu S.Y. (2022). Homologous or Heterologous mRNA Booster Vaccination Induces Robust Neutralizing Antibody Responses Against SARS-CoV-2 Omicron Variant in Individuals Receiving mRNA or Inactivated Virus Priming Vaccination. Lancet.

[34] Mengist H.M., Kombe Kombe A.J., Mekonnen D., Abebaw A., Getachew M., Jin T. (2021). Mutations of SARS-CoV-2 spike protein: Implications on immune evasion and vaccine-induced immunity. Semin. Immunol..

[35] Ivanov K.I., Yang H., Sun R., Li C., Guo D. (2024). The emerging role of SARS-CoV-2 nonstructural protein 1 (nsp1) in epigenetic regulation of host gene expression. FEMS Microbiol. Rev..

[36] Lin J.W., Tang C., Wei H.C., Du B., Chen C., Wang M., Zhou Y., Yu M.X., Cheng L., Kuivanen S. (2021). Genomic monitoring of SARS-CoV-2 uncovers an Nsp1 deletion variant that modulates type I interferon response. Cell Host Microbe.

[37] Davis C., Logan N., Tyson G., Orton R., Harvey W., Haughney J., Perkins J., Peacock T.P., Barclay W.S., The COVID-19 Genomics UK (COG-UK) Consortium (2021). Reduced neutralization of the Delta (B.1.617.2) SARS-CoV-2 variant of concern following vaccination. medRxiv.

[38] Ozkaya E., Yazici M., Baran I., Cetin N.S., Tosun I., Buruk C.K., Kaklikkaya N., Aydin F., Doymaz M.Z. (2023). Neutralization of Wild-Type and Alpha SARS-CoV-2 Variant by CoronaVac(R) Vaccine and Natural Infection-Induced Antibodies. Curr. Microbiol..

[39] Bruxvoort K.J., Sy L.S., Qian L., Ackerson B.K., Luo Y., Lee G.S., Tian Y., Florea A., Aragones M., Tubert J.E. (2021). Effectiveness of mRNA-1273 against delta, mu, and other emerging variants of SARS-CoV-2: Test negative case-control study. BMJ.

[40] Bian L., Gao Q., Gao F., Wang Q., He Q., Wu X., Mao Q., Xu M., Liang Z. (2021). Impact of the Delta variant on vaccine efficacy and response strategies. Expert Rev. Vaccines.

[41] Gupta S.L., Jaiswal R.K. (2022). An Assessment of the Bivalent Vaccine as a Second Booster for COVID-19. Vaccines.

[42] Cao Y., Yisimayi A., Jian F., Song W., Xiao T., Wang L., Du S., Wang J., Li Q., Chen X. (2022). BA.2.12.1, BA.4 and BA.5 escape antibodies elicited by Omicron infection. Nature.

[43] Hachmann N.P., Miller J., Collier A.Y., Ventura J.D., Yu J., Rowe M., Bondzie E.A., Powers O., Surve N., Hall K. (2022). Neutralization Escape by SARS-CoV-2 Omicron Subvariants BA.2.12.1, BA.4, and BA.5. N. Engl. J. Med..

[44] Kimura I., Yamasoba D., Tamura T., Nao N., Suzuki T., Oda Y., Mitoma S., Ito J., Nasser H., Zahradnik J. (2022). Virological characteristics of the SARS-CoV-2 Omicron BA.2 subvariants, including BA.4 and BA.5. Cell.

[45] Tuekprakhon A., Nutalai R., Dijokaite-Guraliuc A., Zhou D., Ginn H.M., Selvaraj M., Liu C., Mentzer A.J., Supasa P., Duyvesteyn H.M.E. (2022). Antibody escape of SARS-CoV-2 Omicron BA.4 and BA.5 from vaccine and BA.1 serum. Cell.

[46] Zou J., Kurhade C., Xia H., Liu M., Xie X., Ren P., Shi P.Y. (2022). Cross-neutralization of Omicron BA.1 against BA.2 and BA.3 SARS-CoV-2. Nat. Commun..

[47] Nguyen N.N., Houhamdi L., Delorme L., Colson P., Gautret P. (2022). Reinfections with Different SARS-CoV-2 Omicron Subvariants, France. Emerg. Infect. Dis..

[48] Ito J., Suzuki R., Uriu K., Itakura Y., Zahradnik J., Kimura K.T., Deguchi S., Wang L., Lytras S., Tamura T. (2023). Convergent evolution of SARS-CoV-2 Omicron subvariants leading to the emergence of BQ.1.1 variant. Nat. Commun..

[49] Tamura T., Ito J., Uriu K., Zahradnik J., Kida I., Anraku Y., Nasser H., Shofa M., Oda Y., Lytras S. (2023). Virological characteristics of the SARS-CoV-2 XBB variant derived from recombination of two Omicron subvariants. Nat. Commun..

[50] Chatterjee S., Bhattacharya M., Dhama K., Lee S.S., Chakraborty C. (2023). Can the RBD mutation R346X provide an additional fitness to the “variant soup,” including offspring of BQ and XBB of SARS-CoV-2 Omicron for the antibody resistance?. Mol. Ther. Nucleic Acids.

[51] Bormann M., Brochhagen L., Alt M., Otte M., Thummler L., van de Sand L., Kraiselburd I., Thomas A., Gosch J., Brass P. (2023). Immune responses in COVID-19 patients during breakthrough infection with SARS-CoV-2 variants Delta, Omicron-BA.1 and Omicron-BA.5. Front. Immunol..

[52] Chen S., Huang Z., Guo Y., Guo H., Jian L., Xiao J., Yao X., Yu H., Cheng T., Zhang Y. (2023). Evolving spike mutations in SARS-CoV-2 Omicron variants facilitate evasion from breakthrough infection-acquired antibodies. Cell Discov..

[53] Kurhade C., Zou J., Xia H., Liu M., Chang H.C., Ren P., Xie X., Shi P.Y. (2023). Low neutralization of SARS-CoV-2 Omicron BA.2.75.2, BQ.1.1 and XBB.1 by parental mRNA vaccine or a BA.5 bivalent booster. Nat. Med..

[54] Tamura T., Yamasoba D., Oda Y., Ito J., Kamasaki T., Nao N., Hashimoto R., Fujioka Y., Suzuki R., Wang L. (2023). Comparative pathogenicity of SARS-CoV-2 Omicron subvariants including BA.1, BA.2, and BA.5. Commun. Biol..

[55] Chye H., Chew C.W.Y., Yeo H.P., Tambyah P.A., Young B.E., Tan G.G.Y., Tan B.H., Vasoo S., Chan C.E.Z. (2023). Neutralization escape of emerging subvariants XBB.1.5/1.9.1 and XBB.2.3 from current therapeutic monoclonal antibodies. J. Med. Virol..

[56] Qu P., Faraone J.N., Evans J.P., Zheng Y.M., Carlin C., Anghelina M., Stevens P., Fernandez S., Jones D., Panchal A.R. (2023). Enhanced evasion of neutralizing antibody response by Omicron XBB.1.5, CH.1.1, and CA.3.1 variants. Cell Rep..

[57] Sugano A., Kataguchi H., Ohta M., Someya Y., Kimura S., Maniwa Y., Tabata T., Takaoka Y. (2023). SARS-CoV-2 Omicron XBB.1.5 May Be a Variant That Spreads More Widely and Faster Than Other Variants. bioRxiv.

[58] Scarpa F., Azzena I., Ciccozzi A., Giovanetti M., Locci C., Casu M., Fiori P.L., Borsetti A., Cella E., Quaranta M. (2023). Integrative Genome-Based Survey of the SARS-CoV-2 Omicron XBB.1.16 Variant. Int. J. Mol. Sci..

[59] Kaku Y., Kosugi Y., Uriu K., Ito J., Hinay A.A., Kuramochi J., Sadamasu K., Yoshimura K., Asakura H., Nagashima M. (2023). Antiviral efficacy of the SARS-CoV-2 XBB breakthrough infection sera against omicron subvariants including EG.5. Lancet Infect. Dis..

[60] Wang Q., Iketani S., Li Z., Liu L., Guo Y., Huang Y., Bowen A.D., Liu M., Wang M., Yu J. (2023). Alarming antibody evasion properties of rising SARS-CoV-2 BQ and XBB subvariants. Cell.

[61] Zhang L., Kempf A., Nehlmeier I., Cossmann A., Dopfer-Jablonka A., Stankov M.V., Schulz S.R., Jack H.M., Behrens G.M.N., Pohlmann S. (2023). Neutralisation sensitivity of SARS-CoV-2 lineages EG.5.1 and XBB.2.3. Lancet Infect. Dis..

[62] Lasrado N., Collier A.Y., Hachmann N.P., Miller J., Rowe M., Schonberg E.D., Rodrigues S.L., LaPiana A., Patio R.C., Anand T. (2023). Neutralization escape by SARS-CoV-2 Omicron subvariant BA.2.86. Vaccine.

[63] Sheward D.J., Yang Y., Westerberg M., Oling S., Muschiol S., Sato K., Peacock T.P., Karlsson Hedestam G.B., Albert J., Murrell B. (2023). Sensitivity of the SARS-CoV-2 BA.2.86 variant to prevailing neutralising antibody responses. Lancet Infect. Dis..

[64] Uriu K., Ito J., Kosugi Y., Tanaka Y.L., Mugita Y., Guo Z., Hinay A.A., Putri O., Kim Y., Shimizu R. (2023). Transmissibility, infectivity, and immune evasion of the SARS-CoV-2 BA.2.86 variant. Lancet Infect. Dis..

[65] Chalkias S., McGhee N., Whatley J.L., Essink B., Brosz A., Tomassini J.E., Girard B., Wu K., Edwards D.K., Nasir A. (2023). Safety and Immunogenicity of XBB.1.5-Containing mRNA Vaccines. medRxiv.

[66] Kaku Y., Okumura K., Padilla-Blanco M., Kosugi Y., Uriu K., Hinay A.A., Chen L., Plianchaisuk A., Kobiyama K., Ishii K.J. (2024). Virological characteristics of the SARS-CoV-2 JN.1 variant. Lancet Infect. Dis..

[67] Cheng S.M.S., Mok C.K.P., Li J.K.C., Chan K.K.P., Luk K.S., Lee B.H.W., Gu H., Chan K.C.K., Tsang L.C.H., Yiu K.Y.S. (2024). Cross-neutralizing antibody against emerging Omicron subvariants of SARS-CoV-2 in infection-naive individuals with homologous BNT162b2 or BNT162b2(WT + BA.4/5) bivalent booster vaccination. Virol. J..

[68] Sohail M.S., Ahmed S.F., Quadeer A.A., McKay M.R. (2024). Cross-Reactivity Assessment of Vaccine-Derived SARS-CoV-2 T Cell Responses against BA.2.86 and JN.1. Viruses.

[69] Li P., Faraone J.N., Hsu C.C., Chamblee M., Zheng Y.M., Carlin C., Bednash J.S., Horowitz J.C., Mallampalli R.K., Saif L.J. (2024). Characteristics of JN.1-derived SARS-CoV-2 subvariants SLip, FLiRT, and KP.2 in neutralization escape, infectivity and membrane fusion. bioRxiv.

[70] Kaku Y., Yo M.S., Tolentino J.E., Uriu K., Okumura K., Genotype to Phenotype Japan C., Ito J., Sato K. (2024). Virological characteristics of the SARS-CoV-2 KP.3, LB.1, and KP.2.3 variants. Lancet Infect. Dis..

[71] Happle C., Hoffmann M., Kempf A., Nehlmeier I., Stankov M.V., Calderon Hampel N., Witte T., Pohlmann S., Behrens G.M.N., Dopfer-Jablonka A. (2024). Humoral immunity after mRNA SARS-CoV-2 omicron JN.1 vaccination. Lancet Infect. Dis..

[72] CDC, IDSA COVID 19 Real-Time Learning Network. https://www.idsociety.org/covid-19-real-time-learning-network/.

[73] Murray S.M., Ansari A.M., Frater J., Klenerman P., Dunachie S., Barnes E., Ogbe A. (2023). The impact of pre-existing cross-reactive immunity on SARS-CoV-2 infection and vaccine responses. Nat. Rev. Immunol..

[74] Yang G., Wang J., Sun P., Qin J., Yang X., Chen D., Zhang Y., Zhong N., Wang Z. (2023). ARS-CoV-2 epitope-specific T cells: Immunity response feature, TCR repertoire characteristics and cross-reactivity. Front. Immunol..

[75] Augusto D.G., Hollenbach J.A. (2022). HLA variation and antigen presentation in COVID-19 and SARS-CoV-2 infection. Curr. Opin. Immunol..

[76] Brand M., Kesmir C. (2023). Evolution of SARS-CoV-2-specific CD4^+^ T cell epitopes. Immunogenetics.

[77] Cohen A.A., Gnanapragasam P.N.P., Lee Y.E., Hoffman P.R., Ou S., Kakutani L.M., Keeffe J.R., Wu H.J., Howarth M., West A.P. (2021). Mosaic nanoparticles elicit cross-reactive immune responses to zoonotic coronaviruses in mice. Science.

[78] Peng L., Renauer P.A., Okten A., Fang Z., Park J.J., Zhou X., Lin Q., Dong M.B., Filler R., Xiong Q. (2022). Variant-specific vaccination induces systems immune responses and potent in vivo protection against SARS-CoV-2. Cell Rep. Med..

[79] Cankat S., Demael M.U., Swadling L. (2024). In search of a pan-coronavirus vaccine: Next-generation vaccine design and immune mechanisms. Cell. Mol. Immunol..

[80] Joyce M.G., Chen W.H., Sankhala R.S., Hajduczki A., Thomas P.V., Choe M., Chang W., Peterson C.E., Martinez E., Morrison E.B. (2021). SARS-CoV-2 ferritin nanoparticle vaccines elicit broad SARS coronavirus immunogenicity. bioRxiv.

[81] Hoffmann M.A.G., Yang Z., Huey-Tubman K.E., Cohen A.A., Gnanapragasam P.N.P., Nakatomi L.M., Storm K.N., Moon W.J., Lin P.J.C., West A.P. (2023). ESCRT recruitment to SARS-CoV-2 spike induces virus-like particles that improve mRNA vaccines. Cell.

[82] Tobias J., Steinberger P., Wilkinson J., Klais G., Kundi M., Wiedermann U. (2024). SARS-CoV-2 Vaccines: The Advantage of Mucosal Vaccine Delivery and Local Immunity. Vaccines.

[83] Lund F.E., Randall T.D. (2021). Scent of a vaccine. Science.

[84] Horton R.E., Vidarsson G. (2013). Antibodies and their receptors: Different potential roles in mucosal defense. Front. Immunol..

[85] Jarlhelt I., Nielsen S.K., Jahn C.X.H., Hansen C.B., Perez-Alos L., Rosbjerg A., Bayarri-Olmos R., Skjoedt M.O., Garred P. (2021). SARS-CoV-2 Antibodies Mediate Complement and Cellular Driven Inflammation. Front. Immunol..

[86] Markiewski M.M., Lambris J.D. (2007). The role of complement in inflammatory diseases from behind the scenes into the spotlight. Am. J. Pathol..

[87] Russell M.W., Moldoveanu Z., Ogra P.L., Mestecky J. (2020). Mucosal Immunity in COVID-19: A Neglected but Critical Aspect of SARS-CoV-2 Infection. Front. Immunol..

[88] Dotiwala F., Upadhyay A.K. (2023). Next Generation Mucosal Vaccine Strategy for Respiratory Pathogens. Vaccines.

[89] Pabst R. (2015). Mucosal vaccination by the intranasal route. Nose-associated lymphoid tissue (NALT)-Structure, function and species differences. Vaccine.

[90] Rathore A.P.S., St John A.L. (2023). Promises and challenges of mucosal COVID-19 vaccines. Vaccine.

[91] Jin G., Wang R., Jin Y., Song Y., Wang T. (2024). From intramuscular to nasal: Unleashing the potential of nasal spray vaccines against coronavirus disease 2019. Clin. Transl. Immunol..

[92] Altay Benetti A., Tan E.Y.Z., Chang Z.W., Bae K.H., Thwin M.T., Muthuramalingam R.P.K., Liao K.-C., Wan Y., Ng L.F.P., Renia L. (2024). Design and Characterization of a New Formulation for the Delivery of COVID-19-mRNA Vaccine to the Nasal Mucosa. Vaccines.

[93] Jakaew P., Jearanaiwitayakul T., Midoeng P., Masrinoul P., Sunintaboon P., Ubol S. (2024). Responses of primary human nasal epithelial cells to COVID-19 vaccine candidate. Asian Pac. J. Allergy Immunol..

[94] Lobaina Y., Chen R., Suzarte E., Ai P., Musacchio A., Lan Y., Chinea G., Tan C., Silva R., Guillen G. (2024). A Nasal Vaccine Candidate, Containing Three Antigenic Regions from SARS-CoV-2, to Induce a Broader Response. Vaccines.

[95] NIH NIH-Sponsored Trial of Nasal COVID-19 Vaccine Opens. https://www.nih.gov/news-events/news-releases/nih-sponsored-trial-nasal-covid-19-vaccine-opens.

[96] Ntziora F., Kostaki E.G., Karapanou A., Mylona M., Tseti I., Sipsas N.V., Paraskevis D., Sfikakis P.P. (2022). Protection of vaccination versus hybrid immunity against infection with COVID-19 Omicron variants among Health-Care Workers. Vaccine.

[97] Ali A., Dwyer D., Wu Q., Wang Q., Dowling C.A., Fox D.A., Khanna D., Poland G.A., Mao-Draayer Y. (2021). Characterization of humoral response to COVID mRNA vaccines in multiple sclerosis patients on disease modifying therapies. Vaccine.

[98] Srivastava K., Carreno J.M., Gleason C., Monahan B., Singh G., Abbad A., Tcheou J., Raskin A., Kleiner G., van Bakel H. (2024). SARS-CoV-2-infection- and vaccine-induced antibody responses are long lasting with an initial waning phase followed by a stabilization phase. Immunity.

[99] Bowman K.A., Stein D., Shin S., Ferbas K.G., Tobin N.H., Mann C., Fischinger S., Ollmann Saphire E., Lauffenburger D., Rimoin A.W. (2022). Hybrid Immunity Shifts the Fc-Effector Quality of SARS-CoV-2 mRNA Vaccine-Induced Immunity. mBio.

[100] Gupta S.L., Jaiswal R.K. (2022). Relevant of neutralizing antibody during SARS-CoV-2 infection and their therapeutic usage. Mol. Biol. Rep..

[101] Keeton R., Tincho M.B., Ngomti A., Baguma R., Benede N., Suzuki A., Khan K., Cele S., Bernstein M., Karim F. (2022). T cell responses to SARS-CoV-2 spike cross-recognize Omicron. Nature.

[102] Khan M.S., Shakya M., Verma C.K. (2024). Exploring immunogenic CD8 + T-cell epitopes for peptide-based vaccine development against evolving SARS-CoV-2 variants: An immunoinformatics approach. VirusDisease.

[103] Aguilar-Bretones M., Fouchier R.A., Koopmans M.P., van Nierop G.P. (2023). Impact of antigenic evolution and original antigenic sin on SARS-CoV-2 immunity. J. Clin. Investig..

[104] Nazaruk P., Tkaczyk I., Monticolo M., Jędrzejczak A.M., Krata N., Pączek L., Foroncewicz B., Mucha K. (2023). Hybrid Immunity Provides the Best COVID-19 Humoral Response in Immunocompromised Patients with or without SARS-CoV-2 Infection History. Vaccines.

[105] Almanzar G., Koosha K., Vogt T., Stein A., Ziegler L., Asam C., Weps M., Schwagerl V., Richter L., Hepp N. (2024). Hybrid immunity by two COVID-19 mRNA vaccinations and one breakthrough infection provides a robust and balanced cellular immune response as basic immunity against severe acute respiratory syndrome coronavirus 2. J. Med. Virol..

[106] Spinardi J.R., Srivastava A. (2023). Hybrid Immunity to SARS-CoV-2 from Infection and Vaccination-Evidence Synthesis and Implications for New COVID-19 Vaccines. Biomedicines.

[107] Bates T.A., Leier H.C., McBride S.K., Schoen D., Lyski Z.L., Lee D.X., Messer W.B., Curlin M.E., Tafesse F.G. (2023). An extended interval between vaccination and infection enhances hybrid immunity against SARS-CoV-2 variants. JCI Insight.

[108] Reeves E.L., Neelam V., Carlson J.M., Olsen E.O., Fox C.J., Woodworth K.R., Nestoridi E., Mobley E., Montero Castro S., Dzimira P. (2024). Pregnancy and infant outcomes following SARS-CoV-2 infection in pregnancy during delta variant predominance—Surveillance for Emerging Threats to Pregnant People and Infants. Am. J. Obstet. Gynecol. MFM.

[109] Greenberg G.C., Vishwakarma N., Tirupattur M.P., Sprague H.M., Katwa L.C. (2023). Implications of COVID-19 Pandemic on Pregnancy: Current Status and Controversies. COVID.

[110] Gunther J., Ziert Y., Andresen K., Pecks U., von Versen-Hoynck F., Network C. (2024). Variability in COVID-19 symptom presentation during pregnancy and its impact on maternal and infant outcomes across the pandemic. Int. J. Infect. Dis..

[111] Jamieson D.J., Rasmussen S.A. (2022). An update on COVID-19 and pregnancy. Am. J. Obstet. Gynecol..

[112] Chaubey I., Vijay H., Govindaraj S., Babu H., Cheedarla N., Shankar E.M., Vignesh R., Velu V. (2023). Impact of COVID-19 Vaccination on Pregnant Women. Pathogens.

[113] Barros F.C., Gunier R.B., Rego A., Sentilhes L., Rauch S., Gandino S., Teji J.S., Thornton J.G., Kachikis A.B., Nieto R. (2024). Maternal vaccination against COVID-19 and neonatal outcomes during Omicron: INTERCOVID-2022 study. Am. J. Obstet. Gynecol..

[114] CDC COVID-19 Vaccination for People Who Are Pregnant or Breastfeeding. https://www.cdc.gov/covid/vaccines/pregnant-or-breastfeeding.html#:~:text=Everyone%20ages%206%20months%20and,pregnancy%20is%20safe%20and%20effective..

[115] Ciapponi A., Berrueta M., Argento F.J., Ballivian J., Bardach A., Brizuela M.E., Castellana N., Comande D., Gottlieb S., Kampmann B. (2024). Safety and Effectiveness of COVID-19 Vaccines During Pregnancy: A Living Systematic Review and Meta-analysis. Drug Saf..

[116] Asirwatham A., Hillman M., Khan L., Sangermano L.M., Leung K., Leftwich H.K. (2024). SARS-CoV-2 Variants in Pregnancy [ID 2683546]. Obstet. Gynecol..

[117] Lam J.N., Nehira J., Phung O., Deng B. (2024). Systematic Review: Safety and Efficacy of mRNA COVID-19 Vaccines in Pregnant Women. J. Pharm. Pract..

[118] Ai J.W., Zhang Y., Zhang H.C., Xu T., Zhang W.H. (2020). Era of molecular diagnosis for pathogen identification of unexplained pneumonia, lessons to be learned. Emerg. Microbes Infect..

[119] Zhou P., Yang X.L., Wang X.G., Hu B., Zhang L., Zhang W., Si H.R., Zhu Y., Li B., Huang C.L. (2020). A pneumonia outbreak associated with a new coronavirus of probable bat origin. Nature.

[120] WHO Diagnostic Testing for SARS-CoV-2: Interim Guidance, 11 September 2020. https://iris.who.int/handle/10665/334254.

[121] Salazar-Ardiles C., Asserella-Rebollo L., Cornejo C., Arias D., Vasquez-Munoz M., Toledo C., Andrade D.C. (2023). Molecular diagnostic approaches for SARS-CoV-2 detection and pathophysiological consequences. Mol. Biol. Rep..

[122] Baba M.M., Bitew M., Fokam J., Lelo E.A., Ahidjo A., Asmamaw K., Beloumou G.A., Bulimo W.D., Buratti E., Chenwi C. (2021). Diagnostic performance of a colorimetric RT -LAMP for the identification of SARS-CoV-2: A multicenter prospective clinical evaluation in sub-Saharan Africa. EClinicalMedicine.

[123] Liu M., Lyu J., Zheng X., Liang Z., Lei B., Chen H., Mai Y., Huang H., Sun B. (2023). Evolution of the newest diagnostic methods for COVID-19: A Chinese perspective. J. Zhejiang Univ. Sci. B.

[124] Gao Y.P., Huang K.J., Wang F.T., Hou Y.Y., Xu J., Li G. (2022). Recent advances in biological detection with rolling circle amplification: Design strategy, biosensing mechanism, and practical applications. Analyst.

[125] Li Y., Yang X., Zhao W. (2017). Emerging Microtechnologies and Automated Systems for Rapid Bacterial Identification and Antibiotic Susceptibility Testing. SLAS Technol..

[126] Uribe-Alvarez C., Lam Q., Baldwin D.A., Chernoff J. (2021). Low saliva pH can yield false positives results in simple RT-LAMP-based SARS-CoV-2 diagnostic tests. PLoS ONE.

[127] Hu X., Deng Q., Li J., Chen J., Wang Z., Zhang X., Fang Z., Li H., Zhao Y., Yu P. (2020). Development and Clinical Application of a Rapid and Sensitive Loop-Mediated Isothermal Amplification Test for SARS-CoV-2 Infection. mSphere.

[128] American Society for Microbiology How the SARS-CoV-2 EUA Antigen Tests Work. https://asm.org/articles/2020/august/how-the-sars-cov-2-eua-antigen-tests-work#:~:text=This%20system%20uses%20chromatogenic%20digital,bind%20to%20the%20N%20antigen..

[129] Nicolai E., Tomassetti F., Pignalosa S., Redi S., Marino M., Basile U., Ciotti M. (2024). The Evolution of Serological Assays during Two Years of the COVID-19 Pandemic: From an Easy-to-Use Screening Tool for Identifying Current Infections to Laboratory Algorithms for Discovering Immune Protection and Optimizing Vaccine Administration. COVID.

[130] Yamayoshi S., Sakai-Tagawa Y., Koga M., Akasaka O., Nakachi I., Koh H., Maeda K., Adachi E., Saito M., Nagai H. (2020). Comparison of Rapid Antigen Tests for COVID-19. Viruses.

[131] Vashist S.K. (2020). In Vitro Diagnostic Assays for COVID-19: Recent Advances and Emerging Trends. Diagnostics.

[132] Kretschmer A., Kossow A., Grune B., Schildgen O., Mathes T., Schildgen V. (2022). False positive rapid antigen tests for SARS-CoV-2 in the real-world and their economic burden. J. Infect..

[133] Ogawa T., Fukumori T., Nishihara Y., Sekine T., Okuda N., Nishimura T., Fujikura H., Hirai N., Imakita N., Kasahara K. (2020). Another false-positive problem for a SARS-CoV-2 antigen test in Japan. J. Clin. Virol..

[134] Barlev-Gross M., Weiss S., Ben-Shmuel A., Sittner A., Eden K., Mazuz N., Glinert I., Bar-David E., Puni R., Amit S. (2021). Spike vs nucleocapsid SARS-CoV-2 antigen detection: Application in nasopharyngeal swab specimens. Anal. Bioanal. Chem..

[135] NIH How Rapid Antigen Tests Perform Against Viral Variants. https://covid19.nih.gov/news-and-stories/how-rapid-antigen-tests-perform-against-viral-variants#:~:text=In%20a%20study%2C%20rapid%20antigen,99%25%20of%20potential%20future%20variants..

[136] Lau K.A., Horan K., Goncalves da Silva A., Kaufer A., Theis T., Ballard S.A., Rawlinson W.D. (2022). Proficiency testing for SARS-CoV-2 whole genome sequencing. Pathology.

[137] Shen Z., Xiao Y., Kang L., Ma W., Shi L., Zhang L., Zhou Z., Yang J., Zhong J., Yang D. (2020). Genomic Diversity of Severe Acute Respiratory Syndrome-Coronavirus 2 in Patients With Coronavirus Disease 2019. Clin. Infect. Dis..

[138] Udugama B., Kadhiresan P., Kozlowski H.N., Malekjahani A., Osborne M., Li V.Y.C., Chen H., Mubareka S., Gubbay J.B., Chan W.C.W. (2020). Diagnosing COVID-19: The Disease and Tools for Detection. ACS Nano.

[139] Chiu C.Y., Miller S.A. (2019). Clinical metagenomics. Nat. Rev. Genet..

[140] Sidiq Z., Hanif M., Dwivedi K.K., Chopra K.K. (2020). Benefits and limitations of serological assays in COVID-19 infection. Indian J. Tuberc..

[141] Guo L., Ren L., Yang S., Xiao M., Chang D., Yang F., Dela Cruz C.S., Wang Y., Wu C., Xiao Y. (2020). Profiling Early Humoral Response to Diagnose Novel Coronavirus Disease (COVID-19). Clin. Infect. Dis..

[142] Gootenberg J.S., Abudayyeh O.O., Lee J.W., Essletzbichler P., Dy A.J., Joung J., Verdine V., Donghia N., Daringer N.M., Freije C.A. (2017). Nucleic acid detection with CRISPR-Cas13a/C2c2. Science.

[143] FDA Sherlock CRISPR SARS-CoV-2 Kit. https://www.fda.gov/media/137747/download.

[144] Li J., Zhang K., Lin G., Li J. (2024). CRISPR-Cas system: A promising tool for rapid detection of SARS-CoV-2 variants. J. Med. Virol..

[145] Chen J., Li Y., Guo L., Zhou X., Zhu Y., He Q., Han H., Feng Q. (2022). Machine learning techniques for CT imaging diagnosis of novel coronavirus pneumonia: A review. Neural Comput. Appl..

[146] Sadeghi A., Sadeghi M., Sharifpour A., Fakhar M., Zakariaei Z., Sadeghi M., Rokni M., Zakariaei A., Banimostafavi E.S., Hajati F. (2024). Potential diagnostic application of a novel deep learning- based approach for COVID-19. Sci. Rep..

[147] Chavda V.P., Valu D.D., Parikh P.K., Tiwari N., Chhipa A.S., Shukla S., Patel S.S., Balar P.C., Paiva-Santos A.C., Patravale V. (2023). Conventional and Novel Diagnostic Tools for the Diagnosis of Emerging SARS-CoV-2 Variants. Vaccines.

[148] Chung Y.S., Lam C.Y., Tan P.H., Tsang H.F., Wong S.C. (2024). Comprehensive Review of COVID-19: Epidemiology, Pathogenesis, Advancement in Diagnostic and Detection Techniques, and Post-Pandemic Treatment Strategies. Int. J. Mol. Sci..

[149] Mohring J., Leithauser N., Wlazlo J., Schulte M., Pilz M., Munch J., Kufer K.H. (2024). Estimating the COVID-19 prevalence from wastewater. Sci. Rep..

[150] Drozdzal S., Rosik J., Lechowicz K., Machaj F., Szostak B., Przybycinski J., Lorzadeh S., Kotfis K., Ghavami S., Los M.J. (2021). An update on drugs with therapeutic potential for SARS-CoV-2 (COVID-19) treatment. Drug Resist. Updat..

[151] Sheahan T.P., Sims A.C., Leist S.R., Schafer A., Won J., Brown A.J., Montgomery S.A., Hogg A., Babusis D., Clarke M.O. (2020). Comparative therapeutic efficacy of remdesivir and combination lopinavir, ritonavir, and interferon beta against MERS-CoV. Nat. Commun..

[152] Focosi D., Franchini M., Maggi F., Shoham S. (2024). COVID-19 therapeutics. Clin. Microbiol. Rev..

[153] Knowlson C., Byrne A., Wilkinson J., Whitmore C., Torgerson D. (2023). The evidence base for emergency use authorizations for COVID-19 treatments: A rapid review. Health Sci. Rep..

[154] Wang P., Nair M.S., Liu L., Iketani S., Luo Y., Guo Y., Wang M., Yu J., Zhang B., Kwong P.D. (2021). Antibody resistance of SARS-CoV-2 variants B.1.351 and B.1.1.7. Nature.

[155] Gottlieb R.L., Nirula A., Chen P., Boscia J., Heller B., Morris J., Huhn G., Cardona J., Mocherla B., Stosor V. (2021). Effect of Bamlanivimab as Monotherapy or in Combination With Etesevimab on Viral Load in Patients With Mild to Moderate COVID-19: A Randomized Clinical Trial. JAMA.

[156] Dougan M., Nirula A., Azizad M., Mocherla B., Gottlieb R.L., Chen P., Hebert C., Perry R., Boscia J., Heller B. (2021). Bamlanivimab plus Etesevimab in Mild or Moderate COVID-19. N. Engl. J. Med..

[157] Baum A., Fulton B.O., Wloga E., Copin R., Pascal K.E., Russo V., Giordano S., Lanza K., Negron N., Ni M. (2020). Antibody cocktail to SARS-CoV-2 spike protein prevents rapid mutational escape seen with individual antibodies. Science.

[158] Weinreich D.M., Sivapalasingam S., Norton T., Ali S., Gao H., Bhore R., Musser B.J., Soo Y., Rofail D., Im J. (2021). REGN-COV2, a Neutralizing Antibody Cocktail, in Outpatients with COVID-19. N. Engl. J. Med..

[159] FDA Authorization (EUA) for Emergency Use of Casirivimab and Imdevimab. https://www.fda.gov/news-events/press-announcements/coronavirus-covid-19-update-fda-authorizes-monoclonal-antibodies-treatment-covid-19.

[160] Westendorf K., Zentelis S., Wang L., Foster D., Vaillancourt P., Wiggin M., Lovett E., van der Lee R., Hendle J., Pustilnik A. (2022). LY-CoV1404 (bebtelovimab) potently neutralizes SARS-CoV-2 variants. Cell Rep..

[161] Liew M.N.Y., Kua K.P., Lee S.W.H., Wong K.K. (2023). SARS-CoV-2 neutralizing antibody bebtelovimab—A systematic scoping review and meta-analysis. Front. Immunol..

[162] Akinosoglou K., Rigopoulos E.A., Kaiafa G., Daios S., Karlafti E., Ztriva E., Polychronopoulos G., Gogos C., Savopoulos C. (2022). Tixagevimab/Cilgavimab in SARS-CoV-2 Prophylaxis and Therapy: A Comprehensive Review of Clinical Experience. Viruses.

[163] Keam S.J. (2022). Tixagevimab + Cilgavimab: First Approval. Drugs.

[164] Zhang X., Yuan H., Yang Z., Hu X., Mahmmod Y.S., Zhu X., Zhao C., Zhai J., Zhang X.X., Luo S. (2022). SARS-CoV-2: An Updated Review Highlighting Its Evolution and Treatments. Vaccines.

[165] Focosi D., Casadevall A. (2022). A Critical Analysis of the Use of Cilgavimab plus Tixagevimab Monoclonal Antibody Cocktail (Evusheld) for COVID-19 Prophylaxis and Treatment. Viruses.

[166] Vellas C., Kamar N., Izopet J. (2022). Resistance mutations in SARS-CoV-2 omicron variant after tixagevimab-cilgavimab treatment. J. Infect..

[167] Cao Y., Wang J., Jian F., Xiao T., Song W., Yisimayi A., Huang W., Li Q., Wang P., An R. (2022). Omicron escapes the majority of existing SARS-CoV-2 neutralizing antibodies. Nature.

[168] Kim C., Ryu D.K., Lee J., Kim Y.I., Seo J.M., Kim Y.G., Jeong J.H., Kim M., Kim J.I., Kim P. (2021). A therapeutic neutralizing antibody targeting receptor binding domain of SARS-CoV-2 spike protein. Nat. Commun..

[169] Ison M.G., Kim J.Y., Sandulescu O., Preotescu L.-L., Martinez N.E.R., Dobryanska M., Birlutiu V., Miftode E.G., Gaibu N., Caliman-Sturdza O.A. (2021). 546. Therapeutic Effect of Regdanvimab in Patients with Mild to Moderate COVID-19: Day 28 Results from a Multicentre, Randomised, Controlled Pivotal Trial. Open Forum Infect. Dis..

[170] Ryu D.K., Kang B., Noh H., Woo S.J., Lee M.H., Nuijten P.M., Kim J.I., Seo J.M., Kim C., Kim M. (2021). The in vitro and in vivo efficacy of CT-P59 against Gamma, Delta and its associated variants of SARS-CoV-2. Biochem. Biophys. Res. Commun..

[171] Syed Y.Y. (2021). Regdanvimab: First Approval. Drugs.

[172] Bock A. (2024). New Guidance Helps Clinicians Use Pemivibart to Protect Immunocompromised Patients From COVID-19. JAMA.

[173] Wang Q., Guo Y., Zhang R.M., Ho J., Mohri H., Valdez R., Manthei D.M., Gordon A., Liu L., Ho D.D. (2023). Antibody neutralisation of emerging SARS-CoV-2 subvariants: EG.5.1 and XBC.1.6. Lancet Infect. Dis..

[174] Roe T.L., Brady T., Schuko N., Nguyen A., Beloor J., Guest J.D., Aksyuk A.A., Tuffy K.M., Zhang T., Streicher K. (2023). Molecular Characterization of AZD7442 (Tixagevimab-Cilgavimab) Neutralization of SARS-CoV-2 Omicron Subvariants. Microbiol. Spectr..

[175] Zhao Q., Wang X., Zhang Z., Liu X., Wang P., Cao J., Liang Q., Qu J., Zhou M. (2023). Serum neutralization of SARS-CoV-2 Omicron BA.2, BA.2.75, BA.2.76, BA.5, BF.7, BQ.1.1 and XBB.1.5 in individuals receiving Evusheld. J. Med. Virol..

[176] Pochtovyi A.A., Kustova D.D., Siniavin A.E., Dolzhikova I.V., Shidlovskaya E.V., Shpakova O.G., Vasilchenko L.A., Glavatskaya A.A., Kuznetsova N.A., Iliukhina A.A. (2023). In Vitro Efficacy of Antivirals and Monoclonal Antibodies against SARS-CoV-2 Omicron Lineages XBB.1.9.1, XBB.1.9.3, XBB.1.5, XBB.1.16, XBB.2.4, BQ.1.1.45, CH.1.1, and CL.1. Vaccines.

[177] Cox M., Peacock T.P., Harvey W.T., Hughes J., Wright D.W., Consortium C.-G.U., Willett B.J., Thomson E., Gupta R.K., Peacock S.J. (2023). SARS-CoV-2 variant evasion of monoclonal antibodies based on in vitro studies. Nat. Rev. Microbiol..

[178] Gupta A., Gonzalez-Rojas Y., Juarez E., Crespo Casal M., Moya J., Rodrigues Falci D., Sarkis E., Solis J., Zheng H., Scott N. (2022). Effect of Sotrovimab on Hospitalization or Death Among High-risk Patients With Mild to Moderate COVID-19: A Randomized Clinical Trial. JAMA.

[179] McCreary E.K., Kip K.E., Collins K., Minnier T.E., Snyder G.M., Steiner A., Meyers R., Borneman T., Adam M., Thurau L. (2022). Evaluation of Bebtelovimab for Treatment of Covid-19 During the SARS-CoV-2 Omicron Variant Era. Open Forum Infect Dis.

[180] Shertel T., Lange N.W., Salerno D.M., Hedvat J., Jennings D.L., Choe J.Y., Brown R.S., Pereira M.R. (2022). Bebtelovimab for Treatment of COVID-19 in Ambulatory Solid Organ Transplant Recipients. Transplantation.

[181] Lilly E. Lilly’s Neutralizing Antibody Bamlanivimab (LY-CoV555) Prevented COVID-19 at Nursing Homes in the BLAZE-2 Trial, Reducing Risk by Up to 80 Percent for Residents. https://investor.lilly.com/news-releases/news-release-details/lillys-neutralizing-antibody-bamlanivimab-ly-cov555-prevented.

[182] Lilly E. Bebtelovimab. https://covid19.lilly.com/.

[183] Arena C.T. Evusheld (Tixagevimab and Cilgavimab) for the Prevention of COVID-19. https://www.clinicaltrialsarena.com/projects/evusheld-tixagevimab-cilgavimab/.

[184] GSK GSK and Vir Biotechnology Announce Sotrovimab (VIR-7831) Receives Emergency Use Authorization from the US FDA for Treatment of Mild-to-Moderate COVID-19 in High-Risk Adults and Paediatric Patients. https://www.gsk.com/en-gb/media/press-releases/gsk-and-vir-biotechnology-announce-sotrovimab-vir-7831-receives-emergency-use-authorization-from-the-us-fda/.

[185] Celltrion. https://www.celltrion.com/en-us.

[186] Invivyd (2024). NVIVYD ANNOUNCES PEMGARDA™ (PEMIVIBART) DEMONSTRATED 84% RELATIVE RISK REDUCTION IN SYMPTOMATIC COVID-19 COMPARED TO PLACEBO IN AN EXPLORATORY ANALYSIS FROM ONGOING CANOPY PHASE 3 CLINICAL TRIAL. https://investors.invivyd.com/news-releases/news-release-details/invivyd-announces-pemgardatm-pemivibart-demonstrated-84-relative/#:~:text=Release%20Details-,Invivyd%20Announces%20PEMGARDA™%20(pemivibart)%20Demonstrated%2084%25%20Relative%20Risk,CANOPY%20Phase%203%20Clinical%20Trial&text=WALTHAM%2C%20Mass.%2C%20Aug.,NEWSWIRE)%20%2D%2D%20Invivyd%2C%20Inc..

[187] Harvey W.T., Carabelli A.M., Jackson B., Gupta R.K., Thomson E.C., Harrison E.M., Ludden C., Reeve R., Rambaut A., Consortium C.-G.U. (2021). SARS-CoV-2 variants, spike mutations and immune escape. Nat. Rev. Microbiol..

[188] Liu H., Wei P., Zhang Q., Chen Z., Aviszus K., Downing W., Peterson S., Reynoso L., Downey G.P., Frankel S.K. (2021). 501Y.V2 and 501Y.V3 variants of SARS-CoV-2 lose binding to bamlanivimab in vitro. MAbs.

[189] Suryadevara N., Shrihari S., Gilchuk P., VanBlargan L.A., Binshtein E., Zost S.J., Nargi R.S., Sutton R.E., Winkler E.S., Chen E.C. (2021). Neutralizing and protective human monoclonal antibodies recognizing the N-terminal domain of the SARS-CoV-2 spike protein. Cell.

[190] Yamasoba D., Kosugi Y., Kimura I., Fujita S., Uriu K., Ito J., Sato K., Genotype to Phenotype Japan C. (2022). Neutralisation sensitivity of SARS-CoV-2 omicron subvariants to therapeutic monoclonal antibodies. Lancet Infect. Dis..

[191] Focosi D., Maggi F., McConnell S., Casadevall A. (2022). Very low levels of remdesivir resistance in SARS-COV-2 genomes after 18 months of massive usage during the COVID19 pandemic: A GISAID exploratory analysis. Antivir. Res..

[192] Sheahan T.P., Sims A.C., Zhou S., Graham R.L., Pruijssers A.J., Agostini M.L., Leist S.R., Schafer A., Dinnon K.H., Stevens L.J. (2020). An orally bioavailable broad-spectrum antiviral inhibits SARS-CoV-2 in human airway epithelial cell cultures and multiple coronaviruses in mice. Sci. Transl. Med..

[193] Vangeel L., Chiu W., De Jonghe S., Maes P., Slechten B., Raymenants J., Andre E., Leyssen P., Neyts J., Jochmans D. (2022). Remdesivir, Molnupiravir and Nirmatrelvir remain active against SARS-CoV-2 Omicron and other variants of concern. Antivir. Res..

[194] Kalil A.C. (2020). Treating COVID-19-Off-Label Drug Use, Compassionate Use, and Randomized Clinical Trials During Pandemics. JAMA.

[195] Liu J., Cao R., Xu M., Wang X., Zhang H., Hu H., Li Y., Hu Z., Zhong W., Wang M. (2020). Hydroxychloroquine, a less toxic derivative of chloroquine, is effective in inhibiting SARS-CoV-2 infection in vitro. Cell Discov..

[196] Yuan Z., Pavel M.A., Wang H., Kwachukwu J.C., Mediouni S., Jablonski J.A., Nettles K.W., Reddy C.B., Valente S.T., Hansen S.B. (2022). Hydroxychloroquine blocks SARS-CoV-2 entry into the endocytic pathway in mammalian cell culture. Commun. Biol..

[197] Alvisi G., Manaresi E., Cross E.M., Hoad M., Akbari N., Pavan S., Ariawan D., Bua G., Petersen G.F., Forwood J. (2023). Importin alpha/beta-dependent nuclear transport of human parvovirus B19 nonstructural protein 1 is essential for viral replication. Antivir. Res..

[198] Caly L., Druce J.D., Catton M.G., Jans D.A., Wagstaff K.M. (2020). The FDA-approved drug ivermectin inhibits the replication of SARS-CoV-2 in vitro. Antivir. Res..

[199] Inada T., Mims C.A. (1985). Ia antigens and Fc receptors of mouse peritoneal macrophages as determinants of susceptibility to lactic dehydrogenase virus. J. Gen. Virol..

[200] Wagstaff K.M., Rawlinson S.M., Hearps A.C., Jans D.A. (2011). An AlphaScreen(R)-based assay for high-throughput screening for specific inhibitors of nuclear import. J. Biomol. Screen.

[201] Zapata-Cardona M.I., Florez-Alvarez L., Guerra-Sandoval A.L., Chvatal-Medina M., Guerra-Almonacid C.M., Hincapie-Garcia J., Hernandez J.C., Rugeles M.T., Zapata-Builes W. (2023). In vitro and in silico evaluation of antiretrovirals against SARS-CoV-2: A drug repurposing approach. AIMS Microbiol..

[202] Liao J., Way G., Madahar V. (2020). Target Virus or Target Ourselves for COVID-19 Drugs Discovery?-Lessons learned from anti-influenza virus therapies. Med. Drug Discov..

[203] Rismanbaf A. (2020). Potential Treatments for COVID-19; a Narrative Literature Review. Arch. Acad. Emerg. Med..

[204] Iketani S., Mohri H., Culbertson B., Hong S.J., Duan Y., Luck M.I., Annavajhala M.K., Guo Y., Sheng Z., Uhlemann A.C. (2023). Multiple pathways for SARS-CoV-2 resistance to nirmatrelvir. Nature.

[205] Beigel J.H., Tomashek K.M., Dodd L.E., Mehta A.K., Zingman B.S., Kalil A.C., Hohmann E., Chu H.Y., Luetkemeyer A., Kline S. (2020). Remdesivir for the Treatment of COVID-19—Final Report. N. Engl. J. Med..

[206] Meyerowitz E.A., Li Y. (2024). Review: The Landscape of Antiviral Therapy for COVID-19 in the Era of Widespread Population Immunity and Omicron-Lineage Viruses. Clin. Infect. Dis..

[207] Mulangu S., Dodd L.E., Davey R.T., Tshiani Mbaya O., Proschan M., Mukadi D., Lusakibanza Manzo M., Nzolo D., Tshomba Oloma A., Ibanda A. (2019). A Randomized, Controlled Trial of Ebola Virus Disease Therapeutics. N. Engl. J. Med..

[208] Gordon C.J., Tchesnokov E.P., Woolner E., Perry J.K., Feng J.Y., Porter D.P., Gotte M. (2020). Remdesivir is a direct-acting antiviral that inhibits RNA-dependent RNA polymerase from severe acute respiratory syndrome coronavirus 2 with high potency. J. Biol. Chem..

[209] Kokic G., Hillen H.S., Tegunov D., Dienemann C., Seitz F., Schmitzova J., Farnung L., Siewert A., Hobartner C., Cramer P. (2021). Mechanism of SARS-CoV-2 polymerase stalling by remdesivir. Nat. Commun..

[210] Agostini M.L., Andres E.L., Sims A.C., Graham R.L., Sheahan T.P., Lu X., Smith E.C., Case J.B., Feng J.Y., Jordan R. (2018). Coronavirus Susceptibility to the Antiviral Remdesivir (GS-5734) Is Mediated by the Viral Polymerase and the Proofreading Exoribonuclease. mBio.

[211] Shannon A., Le N.T., Selisko B., Eydoux C., Alvarez K., Guillemot J.C., Decroly E., Peersen O., Ferron F., Canard B. (2020). Remdesivir and SARS-CoV-2: Structural requirements at both nsp12 RdRp and nsp14 Exonuclease active-sites. Antivir. Res..

[212] Malin J.J., Suarez I., Priesner V., Fatkenheuer G., Rybniker J. (2020). Remdesivir against COVID-19 and Other Viral Diseases. Clin Microbiol. Rev..

[213] Kim S.M., Kim E.H., Casel M.A.B., Kim Y.I., Sun R., Kwak M.J., Yoo J.S., Yu M., Yu K.M., Jang S.G. (2023). SARS-CoV-2 variants with NSP12 P323L/G671S mutations display enhanced virus replication in ferret upper airways and higher transmissibility. Cell Rep..

[214] Stevens L.J., Pruijssers A.J., Lee H.W., Gordon C.J., Tchesnokov E.P., Gribble J., George A.S., Hughes T.M., Lu X., Li J. (2022). Mutations in the SARS-CoV-2 RNA-dependent RNA polymerase confer resistance to remdesivir by distinct mechanisms. Sci. Transl. Med..

[215] Nooruzzaman M., Johnson K.E.E., Rani R., Finkelsztein E.J., Caserta L.C., Kodiyanplakkal R.P., Wang W., Hsu J., Salpietro M.T., Banakis S. (2024). Emergence of transmissible SARS-CoV-2 variants with decreased sensitivity to antivirals in immunocompromised patients with persistent infections. Nat. Commun..

[216] Barnard D.L., Hubbard V.D., Burton J., Smee D.F., Morrey J.D., Otto M.J., Sidwell R.W. (2004). Inhibition of severe acute respiratory syndrome-associated coronavirus (SARSCoV) by calpain inhibitors and beta-D-N4-hydroxycytidine. Antivir. Chem. Chemother..

[217] Painter G.R., Bowen R.A., Bluemling G.R., DeBergh J., Edpuganti V., Gruddanti P.R., Guthrie D.B., Hager M., Kuiper D.L., Lockwood M.A. (2019). The prophylactic and therapeutic activity of a broadly active ribonucleoside analog in a murine model of intranasal venezuelan equine encephalitis virus infection. Antivir. Res..

[218] Reynard O., Nguyen X.N., Alazard-Dany N., Barateau V., Cimarelli A., Volchkov V.E. (2015). Identification of a New Ribonucleoside Inhibitor of Ebola Virus Replication. Viruses.

[219] Gordon C.J., Tchesnokov E.P., Schinazi R.F., Gotte M. (2021). Molnupiravir promotes SARS-CoV-2 mutagenesis via the RNA template. J. Biol. Chem..

[220] Swanstrom R., Schinazi R.F. (2022). Lethal mutagenesis as an antiviral strategy. Science.

[221] Zhao J., Guo S., Yi D., Li Q., Ma L., Zhang Y., Wang J., Li X., Guo F., Lin R. (2021). A cell-based assay to discover inhibitors of SARS-CoV-2 RNA dependent RNA polymerase. Antivir. Res..

[222] Jayk Bernal A., Gomes da Silva M.M., Musungaie D.B., Kovalchuk E., Gonzalez A., Delos Reyes V., Martin-Quiros A., Caraco Y., Williams-Diaz A., Brown M.L. (2022). Molnupiravir for Oral Treatment of COVID-19 in Nonhospitalized Patients. N. Engl. J. Med..

[223] Strizki J.M., Gaspar J.M., Howe J.A., Hutchins B., Mohri H., Nair M.S., Kinek K.C., McKenna P., Goh S.L., Murgolo N. (2024). Molnupiravir maintains antiviral activity against SARS-CoV-2 variants and exhibits a high barrier to the development of resistance. Antimicrob. Agents Chemother..

[224] Sanderson T., Hisner R., Donovan-Banfield I., Hartman H., Lochen A., Peacock T.P., Ruis C. (2023). A molnupiravir-associated mutational signature in global SARS-CoV-2 genomes. Nature.

[225] FDA (2021). FDA Authorizes Additional Oral Antiviral for Treatment of COVID-19 in Certain Adults. https://www.fda.gov/news-events/press-announcements/coronavirus-covid-19-update-fda-authorizes-additional-oral-antiviral-treatment-covid-19-certain.

[226] Zhou S., Hill C.S., Sarkar S., Tse L.V., Woodburn B.M.D., Schinazi R.F., Sheahan T.P., Baric R.S., Heise M.T., Swanstrom R. (2021). beta-d-N4-hydroxycytidine Inhibits SARS-CoV-2 Through Lethal Mutagenesis But Is Also Mutagenic To Mammalian Cells. J. Infect. Dis..

[227] Butler C.C., Hobbs F.D.R., Gbinigie O.A., Rahman N.M., Hayward G., Richards D.B., Dorward J., Lowe D.M., Standing J.F., Breuer J. (2023). Molnupiravir plus usual care versus usual care alone as early treatment for adults with COVID-19 at increased risk of adverse outcomes (PANORAMIC): An open-label, platform-adaptive randomised controlled trial. Lancet.

[228] EMA Lagevrio. https://www.ema.europa.eu/en/medicines/human/EPAR/lagevrio.

[229] Focosi D., McNally D., Maggi F. (2024). The fitness of molnupiravir-signed SARS-CoV-2 variants: Imputation analysis based on prescription counts and GISAID analyses by country. Intervirology.

[230] Hu Q., Xiong Y., Zhu G.H., Zhang Y.N., Zhang Y.W., Huang P., Ge G.B. (2022). The SARS-CoV-2 main protease (M(pro)): Structure, function, and emerging therapies for COVID-19. MedComm.

[231] Jin Z., Du X., Xu Y., Deng Y., Liu M., Zhao Y., Zhang B., Li X., Zhang L., Peng C. (2020). Structure of M(pro) from SARS-CoV-2 and discovery of its inhibitors. Nature.

[232] Ullrich S., Ekanayake K.B., Otting G., Nitsche C. (2022). Main protease mutants of SARS-CoV-2 variants remain susceptible to nirmatrelvir. Bioorg. Med. Chem. Lett..

[233] Hammond J., Leister-Tebbe H., Gardner A., Abreu P., Bao W., Wisemandle W., Baniecki M., Hendrick V.M., Damle B., Simon-Campos A. (2022). Oral Nirmatrelvir for High-Risk, Nonhospitalized Adults with COVID-19. N. Engl. J. Med..

[234] Owen D.R., Allerton C.M.N., Anderson A.S., Aschenbrenner L., Avery M., Berritt S., Boras B., Cardin R.D., Carlo A., Coffman K.J. (2021). An oral SARS-CoV-2 M(pro) inhibitor clinical candidate for the treatment of COVID-19. Science.

[235] Charness M.E., Gupta K., Stack G., Strymish J., Adams E., Lindy D.C., Mohri H., Ho D.D. (2022). Rebound of SARS-CoV-2 Infection after Nirmatrelvir-Ritonavir Treatment. N. Engl. J. Med..

